# Genetic structure of *Micromeria* (Lamiaceae) in Tenerife, the imprint of geological history and hybridization on within‐island diversification

**DOI:** 10.1002/ece3.2094

**Published:** 2016-04-20

**Authors:** Pamela Puppo, Manuel Curto, Harald Meimberg

**Affiliations:** ^1^ CIBIO Research Center in Biodiversity and Genetic Resources/InBio Associated Laboratory University of Porto Campus Vairão Vairão 4485‐661 Portugal; ^2^ Institute for Integrative Nature Conservation Research University of Natural Resources and Life Sciences A‐1180 Vienna Austria

**Keywords:** Genetic structure, hybrid zones, island evolution, Macaronesia, microsatellites, oceanic islands, paleo‐islands, SSR

## Abstract

Geological history of oceanic islands can have a profound effect on the evolutionary history of insular flora, especially in complex islands such as Tenerife in the Canary Islands. Tenerife results from the secondary connection of three paleo‐islands by a central volcano, and other geological events that further shaped it. This geological history has been shown to influence the phylogenetic history of several taxa, including genus *Micromeria* (Lamiaceae). Screening 15 microsatellite markers in 289 individuals representing the eight species of *Micromeria* present in Tenerife, this study aims to assess the genetic diversity and structure of these species and its relation with the geological events on the island. In addition, we evaluate the extent of hybridization among species and discuss its influence on the speciation process. We found that the species restricted to the paleo‐islands present lower levels of genetic diversity but the highest levels of genetic differentiation suggesting that their ranges might have contracted over time. The two most widespread species in the island, *M. hyssopifolia* and *M. varia*, present the highest genetic diversity levels and a genetic structure that seems correlated with the geological composition of the island. Samples from *M. hyssopifolia* from the oldest paleo‐island, Adeje, appear as distinct while samples from *M. varia* segregate into two main clusters corresponding to the paleo‐islands of Anaga and Teno. Evidence of hybridization and intraspecific migration between species was found. We argue that species boundaries would be retained despite hybridization in response to the habitat's specific conditions causing postzygotic isolation and preserving morphological differentiation.

## Introduction

Speciation is traditionally seen as the accumulation of differences between two populations in allopatry, with geographic distance as barrier to gene flow. In general, geneflow will prevent differentiation, so continuous migration and hybridization events will counteract speciation processes (Yeaman and Whitlock 2011) and potentially also homogenize formerly differentiated species when they come secondarily into contact and are not reproductively isolated. However, it had been shown that speciation can occur by adaptation and divergent selection also with geneflow (Seehausen et al. 2014) and several new concepts had been developed that explain the context between genetic diversity, selection, and gene flow, e.g., the hybrid swarm – (Seehausen 2004) or the surfing syngameon hypothesis (Caujapé‐Castells 2011). These hypotheses postulate that populations can work as sink of genetic diversity through hybridization which furthermore could buffer effects of genetic drift and could increase the level of diversity for selection to act upon and could thus foster differentiation by adaptation. This context had become known during the last year as “speciation‐with‐gene‐flow” especially in zoology. A recent paper published by Roy et al. (2015), showed how hybridization in contact zones can transform between‐lineage variation into within‐population genetic diversity increasing the population's potential for adaptation, ultimately favoring adaptive radiations in a short period of time. Overall, hybridization might enhance genetic and phenotypic variation facilitating further divergence and adaptation to changing environmental conditions (Pavarese et al. 2013; Seehausen et al. 2014).

Hybridization might also be able to explain peculiarities of insular radiations, i.e., adaptive evolution on oceanic islands. It can be hypothesized that because of the restricted space available on islands, alleles not under selection might rapidly drift throughout all subpopulations of hybridizing species. In case the selection regime does not stabilize both species, the small ranges will cause the two species to rapidly become one morphospecies. This will be especially pronounced after secondary contact, e.g., by frequent dispersal between current islands or land bridges between paleo‐islands (Puppo et al. 2014, 2015a).

This scenario might explain the comparable high levels of genetic diversity (Pérez de Paz and Caujapé‐Castells 2013; García‐Verdugo et al. 2015). In addition, hybridization can be quite frequent on islands. For example, Kim (2007) found that 34% of the genome in *Sonchus* (Asteraceae) had been exchanged between two species where hybridization has been observed, but the remaining genome had been hypothesized to be stabilized by selection.

Volcanic archipelagos present an ontogeny that is composed of different phases beginning with the growth of a sea mount above the sea level, its continuous building until it reaches its maximum area and height, and its reduction below the sea level by erosion or other catastrophic events such as caldera collapsing and landslides created by volcanic activity (Fernández‐Palacios et al. 2011). This continuous change in profile directly affects speciation opportunities by increasing or diminishing habitat availability as explained by the theory of island biogeography (MacArthur and Wilson 1967) and by the general dynamic model of oceanic island evolution (Whittaker et al. 2007, 2008).

**Figure 1:**
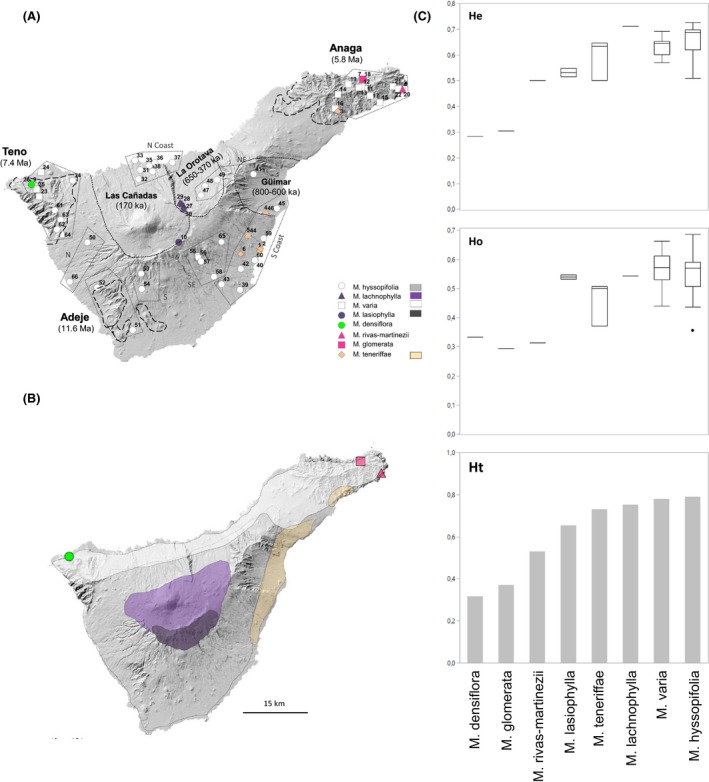
Maps of Tenerife showing: (A) *Micromeria* sampling localities; long‐dashed lines indicate remnants of paleo‐islands; short‐dashed lines indicate major valleys; dotted polygons indicate regions formed by geographically close populations (see Table 1); symbol shapes and colors correspond to different species of *Micromeria*; numbers on symbols indicate collection localities (see Table 1); (B) distribution of *Micromeria* species. Species distributions were obtained by converting a point per quadrant dataset from Pérez de Paz (1978) into continuous ranges. Individuals of *M. varia* on the central north coast had been assigned to *M. hyssopifolia* in the meanwhile (Puppo et al. 2014); (C) genetic diversity for each species calculated as *H*_E_ (upper right), *H*_O_ (middle right), and *H*_T_ (bottom right). The boxplots showing *H*_E_ and *H*_O_ were made from single values estimated per population.

One example of a volcanic archipelago is the Canary Islands, composed of seven islands located ca. 100 km off the western coast of Morocco in the Atlantic Ocean. The islands have each an independent origin, being oldest in the east and youngest toward the west (Carracedo 1994; Juan et al. 2000; Fernández‐Palacios et al. 2011). Among the Canaries, Tenerife presents the most complex geological history and is currently the highest and largest island of the archipelago. Tenerife used to be three islands: Adeje (11.6–3.5 Ma), Teno (6.7–4.5 Ma) and Anaga (6.5–3.6 Ma), that got secondarily connected during the late Miocene—Pliocene due to successive volcanic activity (Ancochea et al. 1990). There is the possibility that Teno and Adeje created their own island but the three island hypothesis is more accepted (i.e., Ancochea et al. 1990; Guillou et al. 2004; Fernández‐Palacios et al. 2011). Tenerife reached its current shape ca. 2 Ma (Ancochea et al. 1990) and parts of the paleo‐islands remain in Tenerife today and exhibit distinct geomorphological and geological characteristics (Fernández‐Palacios et al. 2011; Fig. 1). They also harbor unique floral elements: at least 55 plant species are endemic to at least one paleo‐island (Trusty et al. 2005): 16 on Anaga, 25 on Teno, and 14 on the smallest paleo‐island region, Adeje (Martín et al. 1999). The floristic differences between the paleo‐island regions might have been further intensified by additional volcanic activity and catastrophic landslide events that might have reisolated parts of the island thus disconnecting existing populations (i.e., Mairal et al. 2015; Otto et al. 2016). From the many landslides occurred during the geological history of Tenerife, three massive ones stand out for creating the three major valleys in Tenerife. Güímar in the southeast and La Orotava in the northeast were formed between 800–600 ka and isolated Anaga from the rest of the island (Ancochea et al. 1990; Watts and Masson 1995; Juan et al. 2000; Fig. 1). Likewise, the valley of Las Cañadas in the north‐center was formed less than 200 ka and reisolated Anaga and Teno (Ancochea et al. 1990; Fig. 1). The Teide volcano filled Las Cañadas becoming the highest point of Tenerife today (3718 m; Fig 1).

The geomorphological history of Tenerife has not only had a strong influence on the composition of the regional flora but there are also examples that show its influence on population differentiation within species and potential impact on speciation. Examples are mainly from animals, where haplotype diversity seems correlated with the paleo‐islands with high haplotype divergence between Teno and Anaga (Gübitz et al. 2000; Brown et al. 2006; Macías‐Hernández et al. 2013), though studies with plants are increasing over the last years (i.e., van Hengstum et al. 2012; Rumeu et al. 2014; Jones et al. 2014; Mairal et al. 2015). It had been postulated that this high divergence and patterns of genetic structure are not only explained by the geomorphological history such as secondary contact and reisolation by landslides and lava streams. Rather, the populations have been probably stabilized by selection, with reduced gene flow between genotypes characterized by the haplotypes and the different ecological conditions. For example, in the case of *Gallotia* lizards and *Tarentola* geckos, this is supported by different color patterns and other traits (Gübitz et al. 2000; Brown et al. 2006). In these examples, since differences are being maintained, the selection regime must be stabilizing the different species preventing them from forming a single morphotype.

Furthermore, in geologically complex islands such as Tenerife species ranges previously disrupted by volcanic activity, landslides, and other geological events could have later come into contact forming small‐scale hybrid zones. Hybrid zones usually develop at zones of secondary contact between interbreeding species. In these zones, hybridization could be somewhat frequent, with introgression and backcross probability decreasing in both directions. The occurrence of hybridogenic introgression can be masked when sequence‐based genetic markers are used to investigate the phylogeny of species (Herben et al. 2005). Multilocus investigations on insular species groups are comparably rare, only a few examples exist where dominant marker sets had been used (e.g., Meimberg et al. 2006; Mairal et al. 2015). Codominant markers are the method of choice to investigate genetic structure, gene flow and differentiation between populations because they allow determining the heterozygote state at one locus. Microsatellites or simple sequence repeat (SSR) are loci that show high level of length polymorphisms and constitute the method of choice for population genetic analyses, normally used for within species investigations. For species groups, they are more rarely applied because even though cross species applicability is observed, application can be technically challenging (Barbará et al. 2007). However, if markers can be identified that successfully amplify across a wider range of species, the use of this marker system allows determining geneflow and differentiation between species (González‐Pérez et al. 2009; Sosa et al. 2013; Turini et al. 2014).

In this paper, we are studying the context of geological history and population differentiation using multiple populations of the species of *Micromeria* Benth. on Tenerife, a genus that comprises paleo‐island endemic representatives next to species that are widely distributed on the island. We use a set of 15 microsatellite markers able to cross amplify all *Micromeria* species from Tenerife (Puppo et al. 2015b), to investigate the genetic structure of the species of *Micromeria* present in this island. With this, we aim to understand the diversification process of this genus in Tenerife, in particular, if the genetic structure can be related to the major geological events that occurred on the island. This is of particular interest for the central area species *M. hyssopifolia*,* M. lachnophylla*,* M. lasiophylla*, and *M. varia*. In addition, we investigate the role of hybridization in the evolution of *Micromeria* in Tenerife since natural hybrids had been described for most of the species of the genus occurring in this island. Introgression after hybridization could have combined Teno and Anaga genotypes and could have facilitated the adaptation to the different ecological niches.

The use of codominant markers and the possibility to determine gene flow within species allow outlining different hypotheses about the influence of hybridization on evolutionary patterns on oceanic islands. This will contribute to create a new perspective on speciation dynamics in oceanic islands: an interaction of gene flow and selection driven by geologic and climatic factors might shape evolutionary processes in these systems.

## Materials and Methods

### Study system, DNA isolation and genotyping

*Micromeria* is a genus of the mint family Lamiaceae, subfamily Nepetoideae, and is composed of ca. 54 species distributed in parts of Africa and Asia, the Mediterranean basin and Macaronesia (Bräuchler et al. 2008). *Micromeria* is present in the Canary Islands with 21 species, presenting the highest diversity on Tenerife and Gran Canaria, with 8 and 7 species, respectively (Puppo and Meimberg 2015). In Tenerife, three species are narrowly restricted to the paleo‐islands, one to Teno (*M. densiflora*) and two to Anaga (*M. glomerata* and *M. rivas‐martinezii*). *Micromeria teneriffae* also grows in Anaga but its range extends toward the southeast up to Fasnia and Güímar (Fig. 1). In the paleo‐islands, these four species grow on old rocks and in the southeast, *M. teneriffae* inhabits the coastal desert. In a phylogenetic analysis of multiple nuclear genes and morphometric analysis, the species associated to the paleo‐islands are not only highly morphologically different from those occupying the central area of the island, but are also older (Puppo et al. 2014). Contrary to this, relations among the common species, i.e., those distributed in the younger parts of the island (*M. varia*,* M. hyssopifolia*,* M. lachnophylla*, and *M. lasiophylla*), are less well supported in the phylogeny and further conclusions about their relationships could not be drawn (Puppo et al. 2014). *Micromeria varia* is distributed along the north part of the island from Teno to Anaga, *M. lachnophylla* grows in the central highland of the island above 2000 m, and *M. lasiophylla* is restricted to the southeast rock cliffs of Las Cañadas, above 2000 m (Fig. 1). The species with the widest distribution, *M. hyssopifolia* occurs throughout the island from 0–2000 m and shows a high level of variability growing from costal desert in the south to the pine forest belt and the middle altitude wet regions in the north (Fig. 1). The species inhabiting this central part come into contact in zones where their distributions overlap and it is possible that hybrid zones between all the species exist.

In total, we included 289 samples of *Micromeria* in the present study representing all currently recognized species in Tenerife. Two to twelve individuals were collected in each of the 66 locations sampled (Table S1; Fig. 1). Collection was conducted in Tenerife during the years of 2010 and 2012 and leaves were conserved in silica gel in the field for subsequent DNA analysis.

Dried leaves were ground and DNA was extracted using the Macherey‐Nagel Plant DNA Extraction Kit (Macherey‐Nagel, Düren, Germany) according to the manufacturer's protocol. The 289 samples were amplified with the 16 microsatellite markers developed for *Micromeria* by Puppo et al. (2015b). Each primer was tagged at the 5′‐ end with one of four different universal primers using the M13‐tailed primer method as described in Curto et al. (2013) and Puppo et al. (2015b). The 16 primers were multiplexed in different polymerase chain reactions (PCR) as in Puppo et al. (2015b) using HotStarTaq Plus Master Mix Kit (Qiagen, Valencia, CA). The multiplex primer combination consisted of 4 nmol of each forward primer, 40 nmol of each reverse primer, and the florescent universal primer. The final volume reaction was 10 *μ*L and contained: 5 *μ*L of QIAGEN Multiplex PCR Master Mix (Qiagen), 1 *μ*L of primer mix and 0.5 *μ*L of template DNA (about 40 ng/*μ*L), and 3 *μ*L of water. PCR was performed using the following cycle profile: 95°C for 15 min; 7 cycles of 95°C for 30 sec; touchdown from 58°C to 55°C, decreasing 0,5°C per cycle for 45 sec; 72°C for 30 sec; 25 cycles of 95°C for 30 sec; 55°C for 45 sec; 72°C for 30 sec; 8 cycles of 95°C for 30 sec; 54°C for 45 sec; 72°C for 30 sec; and a final extension step of 60°C for 30 min. Amplification success was confirmed using 2% agarose gels stained with GelRed (Biotium, Hayward, CA). Genotyping was performed with an internal size standard (Genescan‐500 LIZ; Applied Biosystems, Inc., Foster City, CA) in an ABI3130xl automatic sequencer (Applied Biosystems, Inc.). Alleles were called using GeneMapper ver. 4.0 (Applied Biosystems, Inc.). To check for reproducibility of the data, the amplification and scoring of 96 individuals were independently repeated for all primers mixes.

### Data analyses

For the population level analyses, only localities with at least four individuals sampled were considered. To better understand how the estimates vary across the island regions geographically close localities within the same habitat were considered as one population for some analyses (Fig. 1).

Microsatellite quality was evaluated by quantifying the frequency of null alleles and searching for evidence of genotyping errors such as scoring of stuttering bands and large allele drops. This was performed with the program Micro‐Checker (Van Oosterhout et al. 2004) and only populations with at least five individuals with less than 50% missing data for all markers were used. Additionally, we tested if they followed all assumptions from Hardy─Weinberg Equilibrium (HWE) using the program GenAlEx 6.41 (http://biology-assets.anu.edu.au/GenAlEx/).

Genetic diversity per population was estimated by calculating the total number of alleles (*N*), expected and observed heterozygosities (*H*_E_ and *H*_O_), and portion of private alleles. To prevent biases due to population size, the total unbiased *H*_E_ per species and regions was calculated (*H*_T_). Genetic differentiation was estimated by calculating pairwise *F*_ST_, *R*_ST_, and Nei distance; *R*_ST_, to include the information about allele size when using microsatellites in the distance estimate. This allows to have a better perspective of the evolutionary relationships among groups (Balloux and Goudet 2002). The pairwise matrices for genetic differentiation measures were represented by an UPGM dendrogram, calculated using the program NTSys pc (Rohlf 1993). Deviations from Hardy─Weinberg equilibrium (HWE) were estimated for each population. All these statistics were calculated using the program GenAlEx. The existence of changes in population sizes was evaluated with the program BOTTLENECK v. 1.2.02 (Cornuet and Luikart 1997) under the Stepwise Mutation Model (SMM). Since a low number of loci were used, significant deviations from the mutation‐drift equilibrium were calculated using Wilcoxon signed‐rank (Piry et al. 1999).

Analyses of Molecular Variance (AMOVA) were conducted in GenAlEx 6.41 using *R*_ST_ as the measure of differentiation. This was done to access the distribution of genetic variation within and among several species groupings. The different groupings that had been considered are: paleo‐island species versus central species; different species within paleo‐islands; different species within the central region.

Genetic structure between and within species was also investigated using the Bayesian clustering algorithm implemented in the program STRUCTURE ver. 2.3.3 (Hubisz et al. 2009) and using Principal Coordinates Analysis (PCoA) calculated in GenAlEx 6.41. Creating prior decisions of how taxa are structured may lead to circular conclusions. For these reasons, STRUCTURE was run assuming an admixture model of population structure with default settings for inferring alpha and without any location or population priors. Moreover, it was run with and without considering the allele frequencies to be correlated among populations. To determine the number of *K* (unknown) genetic clusters, *K* was set to range from 1 to 15; the program was run as 10 iterations of 500,000 MCMC generations with a burn‐in of 100,000 generations for each *K*. The most likely *K* was selected by analyzing the second‐order rate of change of the posterior probability of the data (DK) between successive *K* values (Evanno et al. 2005) using Structure Harvester v.0.6.9.3 (http://taylor0.biology.ucla.edu/structureHarvester/). Additionally, the suboptimal value of *K* was searched by redoing the DK test without the optimal and smaller values of *K*. This allowed us to investigate more detailed structure signal shown by our data. All 10 iterations were combined using the greedy algorithm from the program CLUMPP (Jakobsson and Rosenberg 2007) For better interpretation of the results, this analysis was performed for three datasets: a first one containing all samples; a second one containing only central species considered by Puppo et al. (2014) as young lineages (*M. varia*,* M. hyssopifolia*,* M. lasiophylla*, and *M. lachnophylla*), and a third one containing only *M. varia* and *M. hyssopifolia*.

We calculated historical and contemporary migrations rates between all species pairs as proxy of gene flow using the programs MIGRATE v3.2.1 (Beerli and Felsenstein 2001) and BAYSASS v3.0 (Wilson and Rannala 2003), respectively. MIGRATE estimates the number of migrants per generation while BAYSASS calculates the portion of individuals originated from the foreigner population. Because of the genetic structure and spatial distance between *M. varia* from Teno and Anaga, these were considered as two distinct groups. Two independent replicates were performed for each analysis and the average migration rate values are presented. For MIGRATE, these migration rate corresponds to the number of individual migrants per generation from the source population. While for BAYSASS, these correspond to the portion of migrant individuals in the sink originating from the source population. We considered a high migration rate to be above 10 individuals per generation for MIGRATE and 10% for BAYSASS in accordance to previous studies (i.e., Bertrand et al. 2014; Conflitti et al. 2014; Peacock et al. 2015).

MIGRATE was run considering the data under the Brownian motion model and implementing a Bayesian search strategy. One long chain was run saving 25,000 generations with sampling increments of 100 generations after a burnin step of 10,000 generations. We defined the maximum prior boundaries of theta and migration rate to be 200 and 1000, respectively. As recommended by Beerli and Palczewski (2010), a static heating scheme was applied with four temperatures of 1, 1.5, 3, and 1 × 10^6^.

Several test runs were performed with BAYSASS to optimize the acceptance rates and the number of generations that should be excluded in the burnin step as recommended in the program's manual. For each run, trace files were saved and analyzed using the program TRACER v1.5.0 (Rambaut and Drummond 2007). In the final analyses, BAYESASS ran for 20,000,000 generations with a burnin of 2,000,000 and sampling increment of 200. The experimental run with the best acceptance rates (below 0.6) had the DeltaA and DeltaF parameter set to 0.4 and DeltaM to 0.1. For this reason, we used these values for the main analyses.

Because some morphological hybrids were found in our sampling, we tested for the likelihood of them being real hybrids by doing a STRUCTURE analyses with the individuals from the same localities in which they were found. With this approach, we expect that hybrid individuals will show an equal assignment to the clusters from the parent species. This result is only considered to be valid if both species are clearly differentiated (*K* = 2). Morphological intermediate individuals were found in the field between *M. densiflora* and *M. varia* in Teno, *M. rivas‐martinezii,* and *M. varia* in Anaga, *M. teneriffae* and *M. varia* in Anaga, and *M. teneriffae* and *M. hyssopifolia* in the south coast. We performed a STRUCTURE analysis for each species pair with the parameters described above.

## Results

### Genetic diversity

From the 16 microsatellite markers included, one (5978) presented low amplification success (<50%), so only 15 SSRs were used for further analysis. The remaining markers comprised between 11 and 25 alleles, giving a total of 273 analyzed alleles. None of the analyzed populations deviated significantly from Hardy─Weinberg equilibrium for most of the loci. A few deviations were indicated with near marginal *P* values (*P* < 0.05) and only for a few loci and single populations. No locus deviated from Hardy─Weinberg equilibrium across the majority of populations meaning that all its assumptions such as neutrality were met. The same was observed the other way around: no population deviated from HWE for most of the loci analyzed (Table S2). There was no evidence of scoring errors and none of the markers constantly showed high frequency of null alleles in the populations analyzed. The 15 loci investigated were therefore retained in the analysis.

Across all populations, mean number of alleles (*N*) varied from 4.20 (*M. densiflora*,* M. lasiophylla*) to 11.27 (*M. lachnophylla*), *H*_O_ from 0.29 (*M. glomerata*) to 0.62 (*M. hyssopifolia*), and *H*_E_ from 0.28 (*M. densiflora*) to 0.71 (*M. lachnophylla*), *H*_T_ from 0.32 (*M. densiflora*) to 0.81 (*M. hyssopifolia*) (Table 1). Expected heterozygosity increased with range size (Fig. 1), i.e., smaller diversity was found in the restricted paleo‐island species and highest diversity was found in the most widespread species *M. lachnophylla*,* M. varia,* and *M. hyssopifolia*. Genetic diversity of populations and groups of populations were generally similar within one species. Slight differences were found in *M. hyssopifolia* which seems to have the highest genetic diversity in the southern coast (*H*_E_ = 0.70, *H*_O_ = 0.62, and *H*_T_ = 0.81). In *M. teneriffae*, the populations from the Southern coast showed slightly lower diversity (*H*_E_ = 0.57, *H*_O _= 0.44, and *H*_T_ = 0.72) than the population from Anaga (*H*_E_ = 0.65, *H*_O_ = 0.51, and *H*_T_ = 0.72). No differences in genetic diversity were found between the two regions (Anaga and Teno) where *M. varia* grows.

**Table 1:** Genetic variation statistics per regions and species of *Micromeria*. This table contains information regarding number of populations (Pops.); average number of individuals (Ind.); average number of alleles (*N*), observed (*H*_O_) and expected heterozygosity (*H*_E_); portion of private alleles (Priv. Al.), and total heterozygosity (*H*_T_)

Region	Anaga				Teno			Adeje	Teide		Southern coast		Northeast	Northwest	Northern coast	Southeast
Species	*M. rivas‐martinezii*	*M. teneriffae*	*M. varia*	*M. glomerata*	*M. densiflora*	*M. hyssopifolia*	*M. varia*	*M. hyssopifolia*	*M. lachnophylla*	*M. lasiophylla*	*M. teneriffae*	*M. hyssopifolia*	*M. hyssopifolia*			
Nr. Pops.	1	1	8	1	1	3	3	2	1	2	2	3	3	1	8	3
Av. Nr. Ind.	11	6	6.5	5	5	6.33	7	9.5	12	4.5	5	5.67	6	6	1.63	8
N	9.8	5.27	5.98	4.27	4.2	6.07	6.42	9.03	11.27	4.2	4.57	5.42	5.44	5.87	–	7.78
Ho	0.31	0.51	0.57	0.29	0.33	0.46	0.57	0.54	0.54	0.54	0.44	0.62	0.59	0.52	–	0.54
He	0.5	0.65	0.64	0.31	0.28	0.63	0.63	0.61	0.71	0.53	0.57	0.7	0.67	0.65	–	0.67
P. Priv. Al.	–	–	0.15	0.13	0.27	0.07	0.11	0.07	0.13	0.1	0.07	0.089	0.111	0.267	–	0.133
Ht	0.53	0.72	0.75	0.37	0.32	0.75	0.76	0.7	0.75	0.65	0.72	0.81	0.8	0.72	0.81	0.75

The number of alleles private to a particular species was generally low (Table 1) and no correlation to species range was obvious. Only in *M. densiflora* and *M. hyssopifolia* from the Northwest, more than 20% of alleles were private. *Micromeria rivas‐martinezii* and *M. teneriffae* from Anaga did not show any private allele. The private alleles found within a species also tended to be rare. For example, only private alleles in *M. densiflora* and *M. lasiophylla* had a frequency within species above 10% not shown. Frequency of the remaining alleles private to a species was below 10% with an average of 3.5%.

Four of the analyzed populations significantly deviated from the mutation‐drift equilibrium (*P* < 0.05) suggesting that they went through a bottleneck event (Table S2). These were the populations from *M. glomerata* and *M. densiflora*, one population from *M. varia* from Teno, and one population from *M. hyssopifolia* from the Southeast.

### Genetic structure

**Figure 2:**
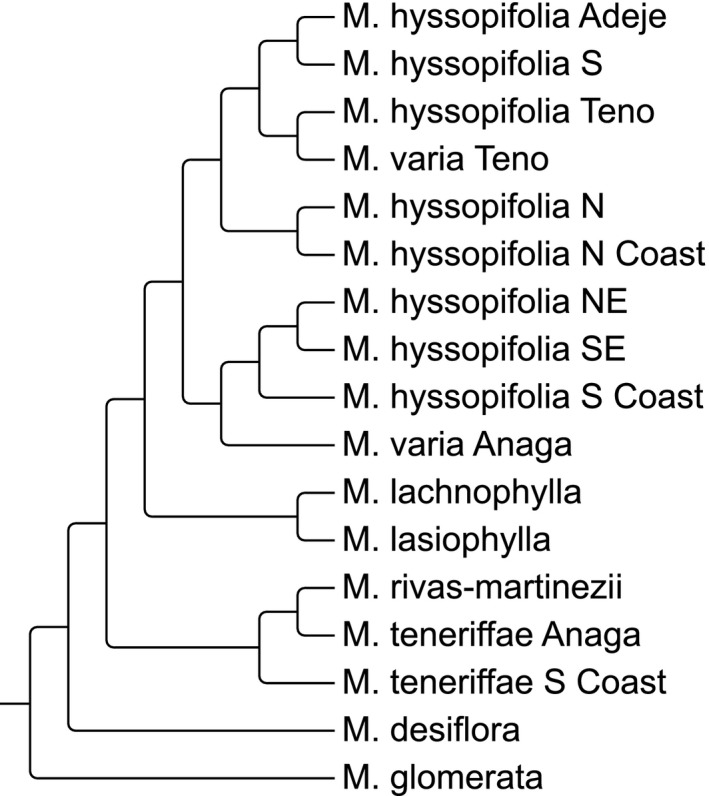
UPGMA of pairwise unbiased uNei distances and RST among population groups of *Micromeria* according to island regions; regions are those showed in Fig 1 and Table 1.

For all populations, the pairwise *F*_ST_ values were highly significant (*P* < 0.001), varying from 0.042 to 0.500 (Table S3). *F*_ST_ was correlated to species age, with the older species (*M. glomerata*,* M. rivas‐martinezii*, and *M. densiflora*) presenting higher pairwise *F*_ST_ values than the youngest (*M. varia* and *M*. *hyssopifolia*). The pairwise unbiased Nei (uNei) distance showed similar patterns to the *F*_ST_ values. *R*_ST_ was calculated among island regions and used to evaluate genetic distance patterns among them (Fig. 2). As expected, the paleo‐island species were the most dissimilar. *Micromeria lasiophylla* and *M. lachnophylla* appear as sister branches to the remaining central species. *M. varia* and *M. hyssopifolia* were mostly grouped according to geographical position. For example, the populations from both species from Teno grouped together.

We performed four independent AMOVA tests using different groupings: all species; paleo‐island species versus central species; different species within paleo‐islands; different species within the central region (Tables 2). The highest amount of variation among groups was explained by differences among paleo‐island species (29%) followed by differences among all species (11%). Difference between paleo‐islands species and central species was explained by 8% of variation. Difference among central species was explained by the lowest amount of variation in the dataset (3%). These results are concordant with the analyses of pairwise *F*_ST_ and *R*_ST_, where higher differentiation is found among paleo‐island species and lower among central species.

**Table 2:** AMOVA analyses of four groupings calculated using *R*_ST_. The results presented in a percentage form correspond to the amount of variation explained by differences within and among groups

Grouping	Number of groups	Number of individuals	Among groups (%)	Within groups (%)
Among all species	8	289	11	89
Among central species	4	245	3	97
Among Paleo‐island species	4	44	29	71
Central species versus Paleo‐island species	2	289	8	92

**Figure 3:**
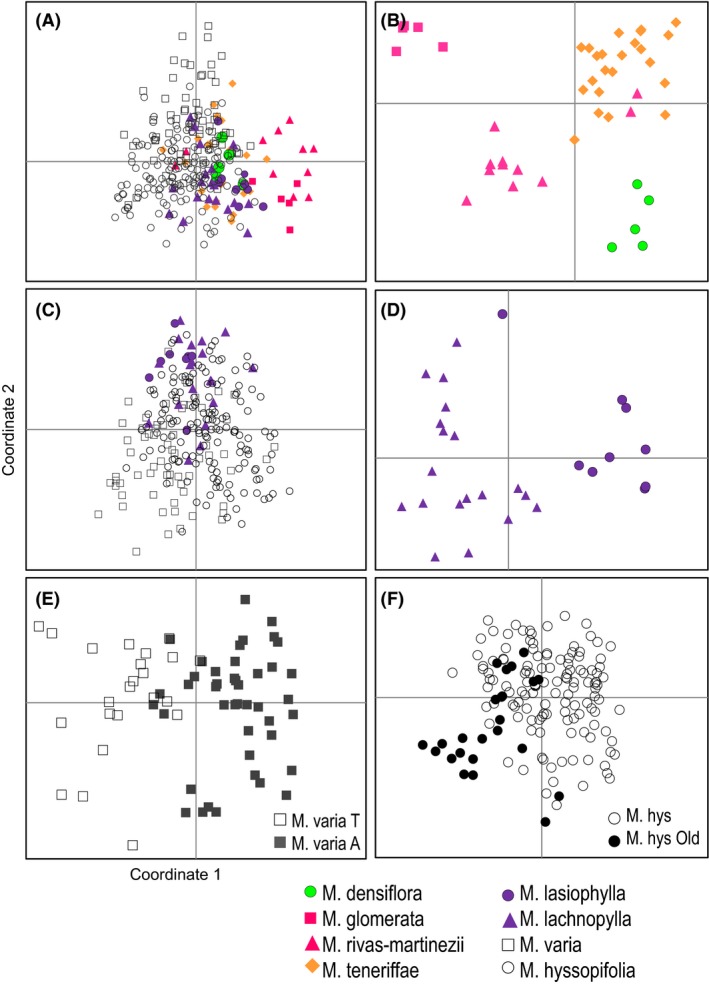
Principal Coordinates Analyses (PCoA) of pairwise distances of individuals of *Micromeria* implemented in GeneAlEx for codominant datasets. Shown are the first two coordinates of analyses including: A. all species; B. only paleo‐island species; C. only central area species; D. only *M. lasiophylla* and *M. lachnophylla*; E. only *M. varia* divided in samples from Anaga (A) and Teno (T); F. only *M. hyssopifolia* divided in samples from Adeje (Old) and the remaining samples.

When pairwise differences are visualized by PCoA, *M. glomerata* and *M. rivas‐martinezii* are separating from the others (Fig. 3A). When only the paleo‐island species are included (*M. teneriffae, M. glomerata*,* M. rivas‐martinezii,* and *M. densiflora*), the PCoA shows four clusters corresponding to each species (Fig. 3B). The analysis including only the central species (*M. lasiophylla*,* M. lachnophylla*,* M. varia,* and *M. hyssopifolia*) shows no separation of the samples (Fig. 3C). When only the central species with narrow range (*M. lasiophylla* and *M. lachnophylla*) are analyzed, there is a distinction among them (Fig. 3D). When *M. varia* is analyzed separately, samples from Anaga slightly segregate from the rest (Fig. 3E). Although the analysis including onl*y M. hyssopifolia* shows no obvious subdivisions of the samples, there is a weak signal of subdivision between individuals located in older and younger parts of the island (Fig. 3F).

**Figure 4:**
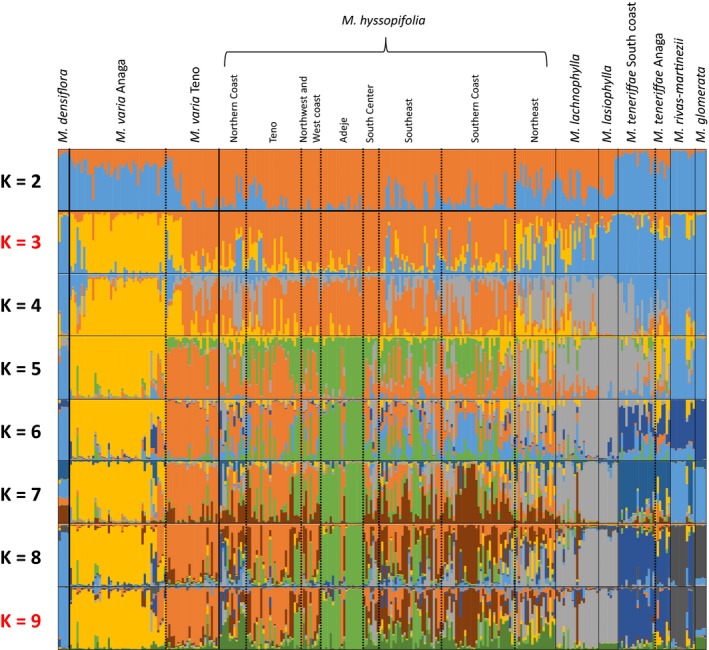
STRUCTURE analyses of the species of *Micromeria* present in Tenerife showing blots of assignment probability from *K* values ranging from *K* = 2 to *K* = 9; optima *K* according to the Evanno method are indicated in red: *K* = 3 for all runs and *K* = 9 when only *K* = 4 to *K* = 15 are analyzed.

**Figure 5:**
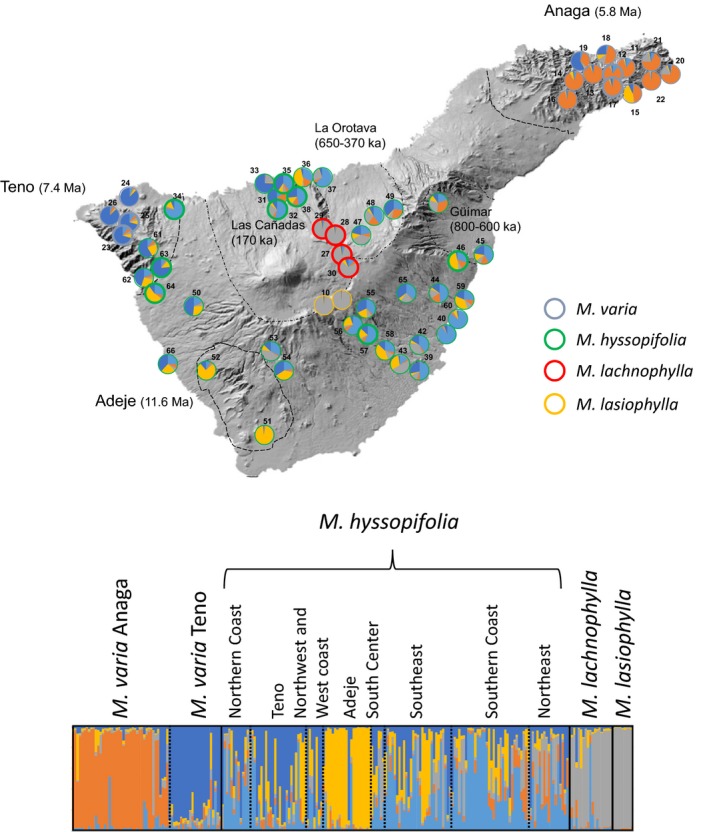
Suboptimum *K* (*K* = 5) for the analyses including only central area species and assignment probability plotted per population on the map. The structure plot is shown to provide a context for the colors shown in the map.

In the STRUCTURE analysis, an optimal *K* = 3 was obtained according to Evanno et al. (2005) method. If results between *K* = 4 and *K* = 15 are tested, optimal *K* is *K* = 9. At *K* = 9, STRUCTURE analysis resolves all species with the exception of *M. lachnophylla* and *M. lasiophylla*. The results from the structure analysis at different values of *K* are summarized in Figure 4. The Delta K plots obtained with STRUCTURE Harvester for all STRUCTURE tests performed are included in Fig. S1. At *K* = 2, *M. varia* from Teno*, M. lachnophylla*,* M. lasiophylla* and *M. hyssopifolia* are forming one of the clusters. At *K* = 3, *M. varia* samples collected in Anaga are forming an additional cluster. At *K* = 5, *M. hyssopifolia* samples from Adeje are forming their own cluster, and with increasing *K*,* M. hyssopifolia* becomes more and more subdivided. When the central species (*M. varia*,* M. hyssopifolia*,* M. lachnophylla* and *M. lasiophylla*) are analyzed independently, this differentiation within *M. hyssopifolia* is clearer. For example, for *K* values higher than 7 one of the clusters is mainly composed of *M. hyssopifolia* samples from the southern coast from subdesert environments, while another cluster is mainly composed of individuals form the wet northern coast. Moreover, samples of *M. hyssopifolia* from Teno share the same cluster with samples of *M. varia* from this same region. The best *K* for the analysis including only the central species was also *K* = 3 and the suboptimum is *K* = 5 (Fig. 5). Although *M. lasiophylla* and *M. lachnophylla* do not separate from each other in these runs, with higher values ok *K* they do. Results were the same for correlated and not correlated allele frequencies, so only analysis with correlated frequencies is shown.

### Gene flow and hybridization

**Figure 6:**
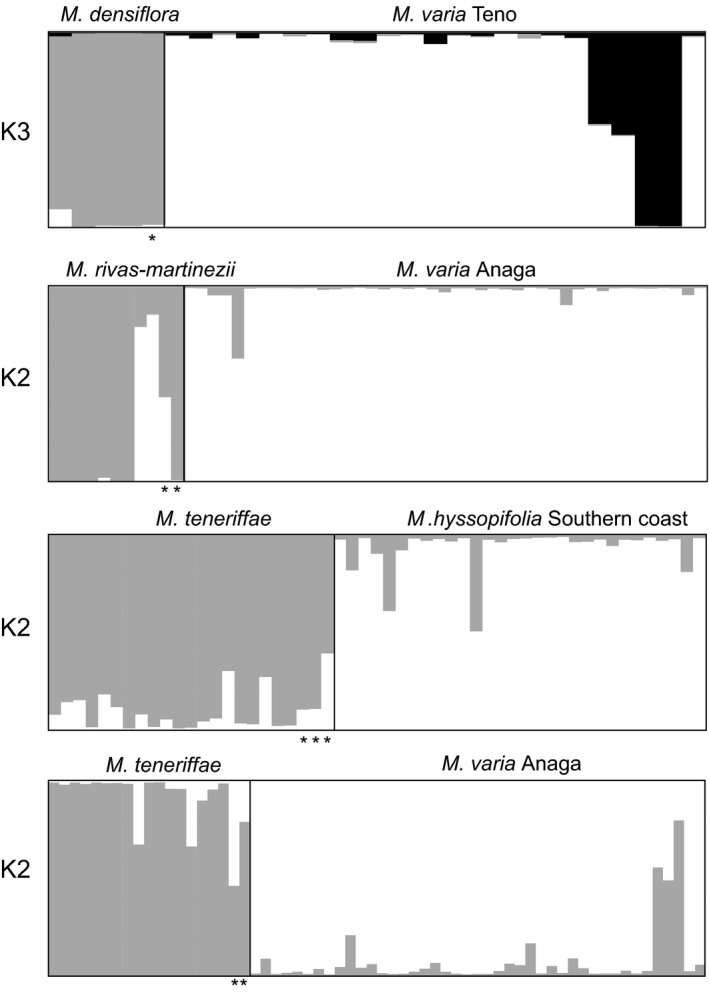
STRUCTURE analyses of potential hybrid individuals of *Micromeria* between four species pairs: *M. densiflora* and *M. varia*;* M. rivas‐martinezii* and *M. varia*;* M. teneriffae* and *M. hyssopifolia* from the South; and *M. teneriffae* and *M. varia* from Anaga. Only the optimal *K* values according to the Evanno method are presented. The individuals marked with * were identified as morphological hybrids.

Several individuals had been determined as hybrids because they present morphologically intermediate characteristics from two species. In a STRUCTURE analysis together with the putative parental species, the hybrid status of most of these individuals were confirmed. According to the DK method, the best *K* was *K* = 3 for the *M. densiflora* and *M. varia* dataset and *K* = 2 for the remaining species pairs (Fig. 6). From the two morphological hybrids between *M. rivas‐martinezii* and *M. varia,* one showed an almost equal assignment to both clusters (44% assignment to *M. varia* cluster), while the other was assigned to the *M. rivas‐martinezii* cluster so it is likely a backcross. In addition to these hybrids, two individuals that were morphologically identified as *M. rivas‐martinezii* showed an almost complete assignment to *M. varia* evidencing introgression between both species. In the analysis between *M. teneriffae* and *M. hyssopifolia*, only one morphological hybrid could be confirmed with high assignment rates to both clusters (39%, of assignment to *M. hyssopifolia* cluster). Two *M. hyssopifolia* individuals showed mixed assignment (41% and 52% to the *M. teneriffae* cluster) suggesting them as hybrids or backcrosses. For the *M. varia* and *M. teneriffae* analysis, only one of the morphological hybrids was confirmed (54% assignment to *M. varia* cluster). Additionally, three *M. varia* individuals showed a high assignment to the *M. teneriffae* cluster (50% to 81%).

BAYSASS and MIGRATE were used to estimate contemporary and historical gene flow among species, respectively. Contemporary gene flow as indicated by BAYSASS was generally low showing migration rates below 10% of individuals originated from other populations for most of the comparisons (Table 3). The exceptions were migration rates from *M. densiflora* to *M. lasiophylla* (17%), *M. glomerata* to *M. rivas‐martinezii* (16%), *M. lachnophylla* to *M. hyssopifolia* (25%), and *M. varia* from Teno to *M. hyssopifolia* (26%).

**Table 3:** Contemporary migration rates calculated by BAYESASS between species of *Micromeria*. Results are presented in percentage (%) of individuals from species on top originated from the species in the left; values for migration within taxa are highlighted in gray; values above 10% are presented in bold; standard deviation values are in parentheses

Source/Sink	*M. densiflora*	*M. glomerata*	*M. hyssopifolia*	*M. lachnophylla*	*M. lasiophylla*	*M. rivas‐martinezii*	*M. teneriffae*	*M. varia* Anaga	*M. varia* Teno
*M. densiflora*	69.44 (2.39)	1.9 (1.8)	1.94 (1.81)	1.93 (1.81)	**16.98 (4.44)**	1.91 (1.81)	2.03 (2.01)	1.94 (1.83)	1.92 (1.81)
*M. glomerata*	1.93 (1.82)	69.46 (2.41)	1.93 (1.83)	1.94 (1.83)	1.93 (1.81)	**15.59 (6.06)**	3.37 (4.98)	1.92 (1.81)	1.93 (1.8)
*M. hyssopifolia*	0.21 (0.21)	0.21 (0.21)	97.78 (0.69)	0.21 (0.21)	0.24 (0.24)	0.39 (0.3)	0.29 (0.29)	0.45 (0.33)	0.22 (0.21)
*M. lachnophylla*	1.01 (0.96)	1.01 (0.96)	**24.92 (2.56)**	67.91 (1.17)	1.02 (0.98)	1.01 (0.97)	1.09 (1.05)	1.01 (0.97)	1.02 (0.97)
*M. lasiophylla*	1.84 (1.73)	1.85 (1.73)	1.86 (1.76)	1.85 (1.76)	84.91 (3.86)	2.04 (1.91)	1.95 (1.85)	1.85 (1.75)	1.86 (1.76)
*M. rivas‐martinezii*	1.64 (1.56)	1.62 (1.54)	1.63 (1.56)	1.63 (1.56)	1.63 (1.56)	81.43 (5.88)	7.14 (6.1)	1.64 (1.56)	1.64 (1.57)
*M. teneriffae*	1.05 (1.01)	1.06 (1.03)	2.46 (1.8)	1.05 (1.01)	1.06 (1.02)	1.06 (1.02)	89.06 (2.96)	2.18 (1.61)	1.04 (1.01)
*M. varia* Anaga	0.64 (0.62)	0.64 (0.63)	0.87 (0.83)	0.65 (0.64)	0.91 (0.81)	0.72 (0.7)	0.78 (0.77)	94.15 (1.82)	0.65 (0.64)
*M. varia* Teno	0.9 (0.87)	0.9 (0.86)	**25.96 (2.3)**	0.9 (0.87)	0.9 (0.86)	0.89 (0.86)	0.91 (0.88)	0.9 (0.87)	67.75 (1.03)

The historical migration rates calculated by MIGRATE varied between 1.4 and 19.1 individuals per generation (Table 4). *Micromeria hyssopifolia* showed to be the main source of interspecific gene flow because it had the highest migration rate (to *M. lachnophylla*). And, from the eight comparisons, five showed migration rates above 10 individuals per generation. *Micromeria varia* was the second main source of migrants, with both *M. varia* from Teno and *M. varia* from Anaga showing three migration comparisons above 10 individuals per generation. *Micromeria rivas‐matrtinezii* was the main sink population because it received more than 10 migrants per generation from six other species. The species with lowest emigration and immigration, less than 10 individuals per generation, were *M. rivas‐martinezii* and *M. glomerata*, respectively. Some loci showed higher values of migration rate than others. On average, the overall migration rate per locus varied from 4.66 individuals per generation for locus 5419 to 175.41 for locus 3963 (not shown).

**Table 4:** Historical migration rates calculated by MIGRATE between species of *Micromeria*. Results are presented in average number of individuals per generation; values correspond to migration rates from the species in the left to the species on top; values for migration within taxa are highlighted in gray; values above 10 migrants per generation are presented in bold; values for 95% confidence intervals are in parentheses

Sorce/Sinck	*M. densiflora*	*M. glomerata*	*M. hyssopifolia*	*M. lachnophylla*	*M. lasiophylla*	*M. rivas‐martinezii*	*M. teneriffae*	*M. varia* Anaga	*M. varia* Teno
*M. densiflora*	–	6.56 (0–23.33)	4.56 (0–21.33)	7.17 (0–24)	8.97 (0–25.33)	**13.55 (0**–**29.33)**	3.42 (0–20)	4.21 (0–20.67)	3.95 (0–20.67)
*M. glomerata*	5.36 (0–22)	–	4.06 (0–20.67)	2.57 (0–18.67)	6.27 (0–23.33)	**10.61 (0**–**26.67)**	**10.46 (0**–**26.67)**	4.07 (0–20.67)	9.42 (0–26.67)
*M. hyssopifolia*	**10.74 (0**–**26.67)**	1.41 (0–18)	–	**19.06 (1.33**–**36.67)**	8.88 (0–27.33)	**14.9 (0**–**30.67)**	3.62 (0–21.33)	**17.66 (0.67**–**34.67)**	**13.11 (0**–**29.33)**
*M. lachnophylla*	4.53 (0–21.33)	5.79 (0–22.67)	6.09 (0–22.67)	–	**14.91 (0**–**30.67)**	5.86 (0–22)	**10.89 (0**–**26.67)**	7.84 (0–24)	6.21 (0–22.67)
*M. lasiophylla*	8.17 (0–24.67)	5.23 (0–22)	5.85 (0–22)	5.97 (0–22.67)	–	9.14 (0–25.33)	5.9 (0–22.67)	**10.96 (0**–**26.67)**	4.89 (0–21.33)
*M. rivas‐martinezii*	6.07 (0–22.67)	3.13 (0–19.33)	4.75 (0–21.33)	8.75 (0–24.67)	4.78 (0–21.33)	–	5.8 (0–22.67)	5.67 (0–22)	6.96 (0–23.33)
*M. teneriffae*	7.48 (0–24)	3.82 (0–20.67)	9.36 (0–25.33)	3.86 (0–20)	**10.19 (0**–**26.67)**	**10.08 (0**–**26.67)**	–	9.99 (0–26)	4.4 (0–21.33)
*M. varia* Anaga	7.19 (0–24)	3.65 (0–20)	**10.72 (0**–**26.67)**	9.14 (0–25.33)	7.94 (0–24.67)	**10.38 (0**–**26.67)**	**10.5 (0**–**26.67)**	–	9.55 (0–26)
*M. varia* Teno	**11.75 (0**–**27.33)**	3.8 (0–20)	**12.64 (0**–**28)**	6.49 (0–23.33)	4.45 (0–21.33)	**13.77 (0**–**29.33)**	9.23 (0–25.33)	8.25 (0–24.67)	–

## Discussion

### Geomorphological impact on genetic structure

In geologically complex islands such as Tenerife, secondary connection of previously isolated parts, successive volcanic activity, caldera collapses, landslides, etc, could have produced a strong impact on the diversification of its species (Whittaker et al. 2007, 2008; Fernández‐Palacios et al. 2011). Several molecular studies in different organisms have found diversification patterns coinciding with the different geological events in Tenerife (e.g., Juan et al. 2000; Carine et al. 2004; Moya et al. 2004; Trusty et al. 2005; Mairal et al. 2015). In *Micromeria*, Puppo et al. (2014) showed that species restricted to the paleo‐islands are early diverging lineages and are older than the central area species. Hereby the restricted ranges of *M. densiflora* from Teno, *M. glomerata* and *M. rivas‐martinezii* from Anaga can be interpreted as contracted ranges, remnants of an earlier, wider distribution, while the range of *M. teneriffae* can be regarded as a shift from Anaga to the surrounding areas after the uprising of the Teide (Puppo et al. 2014). In the present analysis, we found that the highest differentiation is between these four species restricted to the paleo‐islands, which is in accordance to Puppo et al. (2014) phylogenetic hypothesis. The AMOVA results also support this previous study since higher variation was found among paleo‐endemic species than among central species. Since these species are older, they had more time to accumulate genetic differences and are more reproductively isolated. The low differentiation between paleo‐island and non‐paleo‐island species might be explained by the fact that high genetic variation found among paleo‐island species is increasing the variation within groups.

The distance analysis of pairwise RST, is highly congruent with the previous phylogenetic inferences. In both analyses, the paleo‐island species are clustering independently from the central species group. The difference is mainly in the most widespread species: using the microsatellite dataset, they are positioned more pronouncedly according to geography. For example, species from Teno are always clustering together while *M. varia* from Anaga appears together with geographically proximate *M. hyssopifolia* populations. The same is observed for *M. lasiophylla* and *M. lachnophylla* that occur on high altitude in the Teide Mountain. This might be a result of gene flow between the respective populations and is further discussed below.

Genetic diversity of the restricted species was lower than the common species, indicating the possibility that their ranges are contracted. This is supported also by the bottleneck analysis for the populations of *M. densiflora* and *M. glomerata*.

Our study shows that the two most widespread species on the island, *M. varia* and *M. hyssopifolia,* present a genetic structure that is highly correlated to the geological composition of Tenerife. In *M. varia*, samples from Teno and from Anaga are assigned to two different clusters. Samples of *M. hyssopifolia* from Teno cluster together with the *M. varia* samples from this region. This clustering is already indicated in the STRUCTURE analysis when *K* = 2 and is also evident in the PCoA. Additionally, the optimal division in STRUCTURE corresponds to the appearance of a unique cluster of *M. varia* from Anaga showing that this corresponds to a deep divergence. *Micromeria varia* is assumed to be distributed along the northern part of Tenerife from Teno to Anaga. However, samples from the central part of the northern coast have been identified as a different subspecies of *M. hyssopifolia*, subsp. *glabrescens* (sensu Pérez de Paz 1978). Therefore, *M. varia* might be restricted to the paleo‐islands. Hence, the genetic structure observed might be either a consequence of the ancestral split of the two paleo‐islands or a consequence of the reisolation of Anaga after the central shield was formed. For example, Anaga was reisolated by several events such as two massive landslides in the north of Tenerife: one occurred ca. 650–370 ka giving origin to La Orotava valley, the second ca. 170 ka formed Las Cañadas Caldera (Ancochea et al. 1990; Watts and Masson 1995; Juan et al. 2000). The populations of *M. varia* from these two paleo‐islands might have been isolated since then. In our previous work (Puppo et al. 2014), we found that *M. varia* from Anaga was grouped together with the older lineages resulting in a separation from Teno before these landslides. This was assumed to be a consequence of hybridization of *M. varia* populations with the other Anaga species. However, now more Anaga populations are included and all show the same pattern. Other events might have contributed to the isolation of both *M. varia* groups. As in *M. varia*, the divergence between Teno and Anaga populations has been observed in at least two other plant species, *Hypericum canariense* (Clusiaceae; Dlugosch and Parker 2007) and *Canarina canariensis* (Campanulaceae, Mairal et al. 2015), and also in studies of mitochondrial haplotype diversity in several animal groups (e.g., Gübitz et al. 2000; Brown et al. 2006). It had been suggested that this difference stems from habitat discontinuities between and within paleo‐islands that causes strong divergent selection and impedes migration (Gübitz et al. 2000; Moya et al. 2004).

Similar to the structure observed within *M. varia*, genetic divergence within *M. hyssopifolia* seems also related to the paleo‐islands, in particular since these samples were assigned to multiple clusters in the STRUCTURE plot. Especially evident is the segregation of the individuals from Adeje which is the oldest paleo‐island. Differently from Teno and Anaga which are forming rather independent shields, the remnant of Adeje is to a higher extent incorporated into the central massif. Our data show that even though secondary contact of Adeje and Teide central massif is supposed to be around 2 million years ago (Ancochea et al. 1990; Cantagrel et al. 1999), the imprint in genetic structure can still be observed. This is the case for the samples from *M. hyssopifolia* collected in Adeje which form a distinct cluster in the STRUCTURE analyses. This can be either explained by Adeje as origin of *M. hyssopifolia*, by different conditions that favors certain genotypes by selection, or recent volcanic events that kept these populations isolated.

### Hybrid zones and potential ecological effects

Our analysis indicates a strong influence of historical and contemporary gene flow between the species on the genetic structure, most pronouncedly in *M. hyssopifolia*. Hybridization between different *Micromeria* species in Tenerife is well documented and hybrids between most of the species have been described: *M. varia × teneriffae, M. varia × rivas‐martinezii, M. varia × densiflora, M. varia × M. glomerata, M. teneriffae × hyssopifolia* (Pérez de Paz 1978; Santos‐Guerra et al. 2011). Some of these individuals were included in our dataset and their status as hybrids were confirmed: *M. varia × rivas‐martinezii, M. varia × teneriffae, M. teneriffae × hyssopifolia* because they show genotypes intermediate between the parent species. For the two first species pairs, these intermediate genotypes were found in individuals morphologically not classified as hybrids suggesting that they might be backcrosses. The respective two individuals were collected in the contact zone between *M. varia* and *M. rivas‐martinezii* populations where both species grow together (Puppo pers. obs.). Three samples of *M. varia* growing in this contact zone were also assigned to *M. teneriffae*, which might be a consequence of introgression of ancestral alleles shared by *M. teneriffae* and *M. rivas‐martinezii*.

A lower degree of reproductive isolation between island species, compared to continental ones, is generally assumed because of a potentially comparable lower effect of fitness decrease after hybridization resulting from the lower levels of interspecific competition in island systems (Herben et al. 2005). This context had been discussed in several studies and reviews (i.e., Thomas and Leggett 1974; Charmet et al. 1996; Herben et al. 2005; Silvertown et al. 2005).

In Tenerife, species of *Micromeria* have a pronounced allopatric distribution, i.e., species do not occur in sympatry but only come into contact in relatively small areas where ranges overlap (Fig. 1). Is in these contact zones where hybridization occurs. There are two possible explanations for this distributional pattern. Species might either have evolved in parapatry (Gavrilets et al. 2000) where edge populations differentiate from a larger central population, i.e., in populations of *M. lachnophylla*/*M. lasiophylla* and populations of *M. varia* from Teno. Or, species ranges might have developed after secondary contact of well differentiated species after merging of the paleo‐islands. In any case, even in the presence of hybridization, species boundaries are maintained due to differential local selective pressures causing postzygotic isolation and preservation of morphological differences (Seehausen et al. 2014). This typically leads to a hybrid zone dynamic (Barton and Hewitt 1985). Via backcrossing alleles at neutral loci can pass the hybrid zone in both directions, while loci under strong selection cannot and form the base for species specific differences in morphology and ecology. This differential introgression pattern is very well studied and regarded as a typical expression of the contact zone between two species that are able to form fertile hybrids (i.e., Teeter et al. 2010; Nosil et al. 2012; Larson et al. 2014). An example of how selection favors certain genotypes in dependence of the ecological zone is the gecko *Tarentola delallandii* (Gübitz et al. 2000). Despite being the same species, three highly distinct mitochondrial haplotypes originated from the three paleo‐islands. This means that, despite the current contact zone, and being the same species, gene flow between the corresponding groups might be low.

Besides the tests for migration, the existence of hybrid zones between the allopatric ranges of the species is supported in our study by three main findings: (1) we observed and verified the status of hybrids in the contact zones of four species pairs; (2) the two species with the largest contact zones, *M. hyssopifolia* and *M. varia*, show also the highest interspecific migration rates. (3) With exception of *M. densiflora* and *M. lasiophylla,* all other connections through gene flow were indicated between species that have contacting ranges; and (4) cluster arrangement in the structure analysis gives increased assignment probability for adjunct populations even though they belong to different species, e.g., for *M. hyssopifolia* and *M. varia* from Teno and *M. hyssopifolia* and *M. lachnophylla*. Hereby, some loci show higher values for migration than others indicating asymmetric introgression at some degree.

The formation of distinct hybrid zones could be observed directly between *M. varia* and *M. rivas‐martinezii*. Here, in a very small spatial scale hybridization occurs at the transition from the range of *M. rivas‐martinezii* to *M. varia*. *Micromeria rivas‐martinezii* grows in a very restricted area in a small peninsula in the Anaga massif (Hernández‐Pacheco et al. 1990) where *M. varia* does not occur. In a few 100 m wide zone at the main island adjacent to the peninsula, *M. varia × M. rivas martinezii* hybrids occur in small individual numbers that are giving way to morphological *M. varia* populations (Puppo pers. obs.). This transition can also be seen in our SSR data, indicating a transition in the allele frequency content between *M. rivas‐martinezii* and adjacent *M. varia* populations more gradual than expected if the species were reproductively isolated.

The formation of hybrid zones may have contributed to the increase of genetic variation of some taxa facilitating adaptation to changing conditions, shift of ecological niches, or range shift for the species after secondary contact of the paleo‐islands. An example would be *M. hyssopifolia*, which is the species with the largest range. It participates in most of gene‐flow exchanges found in the island and it has one of the highest genetic diversity. Environmental conditions across the range of *M. hyssopifolia* are highly heterogeneous. The northern part of Tenerife is wetter due to the fog brought by the trade winds with high levels of rainfall (ca. 1000 mm precipitation per year) in the mid altitudes. Contrary to this, the southern part of the island is dry (below 100 mm precipitation per year) due to the shade effect caused by the Teide. As described below, this genetic structure might reflect these environmental differences, such as structure found between the wet northern and dry southern slopes. Like outlined above, we see the population from Adeje slightly differentiated from the remaining *M. hyssopifolia* populations. Besides this, at optimal (*K* = 3) and higher *K* (up to *K* = 9), we observed genetic structure among: (1) Teno and west Tenerife; (2) north coast, and (3) southeast and south coast, corresponding to a medium, high and very low precipitation regime. It seems likely that genotypes are locally adapted to these different habitats and genotypes from the southern part may not be able to establish in the northern part and vice versa. These different habitats correspond roughly to the subdivision of *M. hyssopifolia*. Three varieties are recognized within this species*: var. hyssopifolia*,* var. glabrescens,* and *var. kuegleri* (Pérez de Paz 1978) reflecting its morphological diversity. The typical *M. hyssopifolia* (*var. hyssopifolia*) presents a strigose indumentum which gives the plants a grayish aspect and is distributed in the pine forest between 400–2000 m. *Micromeria hyssopifolia var. glabrescens* is mostly distributed in the north of the island in degraded areas between 300–600 m while *var. kuegleri* is the coastal form that grows in the southeast from the sea level up to 400 m. Thus, our structure pattern differentiates mostly *var. glabrescens* and *var. kuegleri*. Because the environmental conditions are not independent from geography, further work is currently being conducted to confirm the hypotheses that: hybridization after secondary contact of former paleo‐island species allowed the colonization of the whole island by one or a few species, and the genetic structure that can be observed in *M. hyssopifolia* is an expression of local adaptation patterns rather than geography.

### Low genetic differentiation levels and microsatellites

The pattern of hybridization found in our study might also explain the apparent low genetic distance between the species with microsatellite datasets and with our earlier multigene analyses (Puppo et al. 2014, 2015a). In Puppo et al. (2015a), low genetic differentiation and low tree resolution were not only found for the central species of Tenerife but also for the most widespread species from Gran Canaria. Because they are usually neutral and have a high mutation rate, microsatellites are frequently used in population genetic studies to identify genetic diversity levels and population differentiation within species but they are rarely used in investigations that cover multiple species (Barbará et al. 2007). Recent examples are Bonatelli et al. (2014) and Turini et al. (2014), where SSR markers and Bayesian clustering had been used to test species boundaries or to establish a species concept.

Gene flow between the species would impact genetic distance. In the case of *Micromeria*, the age estimate especially for the paleo‐island species would suggest that alleles are highly diverged, and the amount of private alleles within one species should be rather high. Even though we found private alleles for the different species, only few of them have within species frequencies above 10%, and most of them are rare alleles. In addition, pairwise *F*_ST_ between populations is only slightly higher between species than within species. Using a microsatellite dataset to investigate different species is likely to underestimate genetic distances between species when hybridization occurs, not only because of shared alleles but also because of the choice of loci during the screen for markers (Turini et al. 2014). With hybridization between species at a contact zone, screen is likely to be biased toward markers that are not linked to loci that are highly structured but to neutral loci that can pass the hybrid zone. We assume therefore that the degree of genetic differentiation between species might be underestimated using a dataset like this. This is especially true when we consider the high morphological distinctness of the species under investigation (Puppo et al. 2014). However, considering introgression and selection for alleles that are exchanged between species, *F*_ST_ below 0.1 could be plausible also between these morphologically highly differentiated species. This effect may also lead to overestimation of migration rates. Nevertheless, this would affect all measures in the same way and not influence interpretations that are made comparatively.

### Phylogeographic and taxonomic considerations

Currently, there are eight species of *Micromeria* recognized in Tenerife with different levels of morphological differentiation. Recent phylogenetic analyses (Puppo et al. 2014) suggest that the genus was probably present in Anaga around 6.7 Ma, before the central shield was formed, and had a first diversification event that gave origin to *M. teneriffae*, and afterwards to *M. glomerata* and *M. rivas‐martinezii*. A second diversification event probably took place in Teno giving origin to *M. densiflora* ca. 4.5 Ma. These four species are also today clearly related to the paleo‐islands. According to this phylogeny, Teno colonized the central part of Tenerife where the remaining four species originated. These analyses were inconclusive with regard to the central species however, since relations among the species were poorly resolved (Puppo et al. 2014). Nevertheless, phylogenetic reconstruction seems to support a scenario where progressive adaptation to higher altitudes of *M. varia* gave origin to *M. hyssopifolia,* and this to *M. lachnophylla* and *M. lasiophylla* (Pérez de Paz 1978; Puppo et al. 2014).

Microsatellite analysis conclusively supports all species when we consider the formation of distinct clusters in the structure analysis. As explained above, it seems likely that hybridization between species is decreasing pairwise differences between the species. In addition, the paleo‐island species appear to a higher extent differentiated from the others and microsatellite analyses provide new insights into the genetic structure of the central species. Interestingly, *M. lasiophylla* is showing close affinities to *M. teneriffae* in an analysis of Nei genetic distances as well as cluster together with the paleo‐islands species for *K* = 2. Even though *M. lasiophylla* and *M. lachnophylla* are not early diverging lineages as the paleo‐island species, this indicates that diversification might precede the secondary contact that occurred ca. 2 Ma ago (Ancochea et al. 1990; Cantagrel et al. 1999). Both *M. lasiophylla* and *M. lachnophylla* grow in old rocks of volcanic origin. It is possible that progenitors of these species colonized from the paleo‐islands independently from the other species instead of being the high altitude forms of *M. varia* or *M. hyssopifolia* as suggested by morphology and phylogenetic analysis (Pérez de Paz 1978; Puppo et al. 2014). In fact, it has been observed in several groups (i.e., Thorpe et al. 1994; Dlugosch and Parker 2007; Cox et al. 2012; Macías‐Hernández et al. 2013) that taxa from Tenerife's paleo‐islands colonized the central, younger part of the island, as well as other younger nearby islands, following a stepping‐stone model (Kimura and Weiss 1964).

The PCoA shows *M. lasiophylla* and *M. lachnophylla* as distinct when analyzed separately from the rest of species (Fig. 3D). When *K* is increased (i.e., *K* = 10), some admixture between *M. lachnophylla* and *M. hyssopifolia* is found. This is probably caused by hybridization with *M. hyssopifolia* since *M. lachnophylla* is distributed from the high desert in Las Cañadas down to the border of the pine forest where *M. hyssopifolia* grows. Furthermore, morphologically intermediate individuals have been reported in several localities that constitute contact zones where both species occur (Pérez de Paz 1978).

## Conflict of Interest

None declared.

## Data Accessibility

Microsatellite data matrix is deposited in Demiurge as: Puppo P, Curto M, Meimberg H (2015a, 2015b) D‐NMICR‐99 http://www.demiurge-project.org/matrix_digests/D-NMICR-99

## Supporting information


**Figure S1**. Delta *K* plots obtained by STRUCTURE Harvester for all STRUCTURE tests performed.

**Table S1.** List of *Micromeria* samples used in the present study including region, locality name and number, geographical coordinates (Latitude, Longitude), number of samples per locality (N), and collection information. TFC, Herbarium of the Universidad de la Laguna in Tenerife.

**Table S2.** Results for HWE and Bottleneck test per population. Here, we present the number of loci deviating from HWE and the *P*‐value for deviations from the mutation‐drift equilibrium (Bottleneck).

**Table S3.** List of pairwise Fst and unbiased Nei distance results for all populations with at least four individuals.
