# Supplementary figures and images for: Genetic structure of Micromeria (Lamiaceae) in Tenerife, the imprint of geological history and hybridization on within‐island diversification
Source: Ecol Evol. 2016 Apr 20;6(11):3443–60. doi: 10.1002/ece3.2094 (PMC5513284; doi:10.1002/ece3.2094)

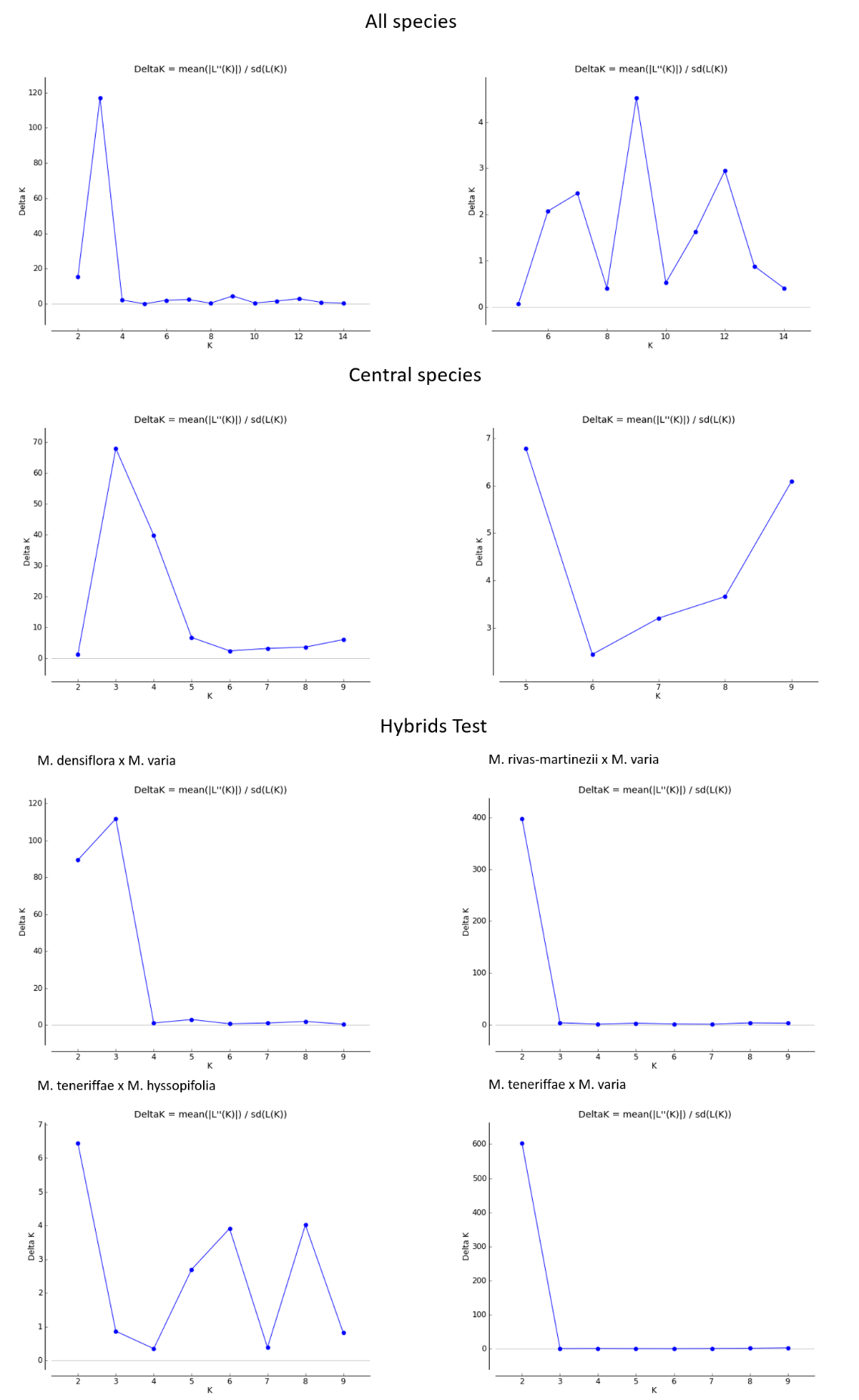
**Fig. S1** Delta K plots obtained by STRUCTURE Harvester for all STRUCTURE tests performed.

Supplement: Supplementary file 1 — Figure S1. Delta K plots obtained by STRUCTURE Harvester for all STRUCTURE tests performed. [file ECE3-6-3443-s001.docx]
