# Supplementary material for: Genetic structure of Micromeria (Lamiaceae) in Tenerife, the imprint of geological history and hybridization on within‐island diversification
Source: Ecol Evol. 2016 Apr 20;6(11):3443–60. doi: 10.1002/ece3.2094 (PMC5513284; doi:10.1002/ece3.2094)
Supplement: Supplementary file 2 — Table S1. List of Micromeria samples used in the present study including region, locality name and number, geographical coordinates (Latitude, Longitude), number of samples per locality (N), and collection information. TFC, Herbarium of the Universidad de la Laguna in Tenerife. [file ECE3-6-3443-s002.docx]

**Table S1** List of *Micromeria* samples used in the present study including region, locality name and number, geographical coordinates (Latitude, Longitude), number of samples per locality (N), and collection information. TFC, Herbarium of the Universidad de la Laguna in Tenerife. Numbers of localities correspond to those in Fig. 1.

| **Species** | **Region** | **Locality** | **Loc. Nr.** | **Latitude** | **Longitude** | **N** | **Collection** |
| --- | --- | --- | --- | --- | --- | --- | --- |
| *M. teneriffae var. cordifolia* | Southern Coast | Fasnia | 1 | 28,2189 | -16,4153 | 4 | Puppo 299-301, 303 (TFC) |
| *M. teneriffae var. cordifolia* | Southern Coast | Fasnia | 2 | 28,22351 | -16,41321 | 3 | Meimberg 25 |
| *M. teneriffae var. teneriffae* | Southern Coast | Güímar | 4 | 28,2944 | -16,403 | 3 | Puppo 151, 154, 156 (TFC) |
| *M. teneriffae var. teneriffae* | Southern Coast | Btw Fasnia-Güímar | 5 | 28,2497 | -16,4382 | 4 | Puppo 160-161, 163-164 (TFC) |
| *M. teneriffae var. teneriffae* | Southern Coast | Bco Eras | 6 | 28,2129 | -16,4532 | 2 | Puppo 166, 168 (TFC) |
| *M. teneriffae var. teneriffae* | Anaga | Bco Tahodio, Anaga | 3 | 28,4982 | -16,2589 | 7 | Puppo 185-188, 190-192 (TFC) |
| *M. glomerata** | Anaga | Taganana, Anaga | 7 |  |  | 5 | Puppo 200-203 (TFC) |
| *M. rivas-martinezii** | Anaga | Roque Juan Bay, Anaga | 8 |  |  | 11 | Puppo 208-215, 218, 227-228 (TFC) |
| *M. densiflora** | Teno | Bujame, Teno | 9 |  |  | 5 | Puppo 255-257, 259-260 (TFC) |
| *M. lasiophylla** | Teide | Teide Nat. Park | 10 |  |  | 9 | Puppo 274-276, 279-281, 283, 286, 288 (TFC) |
| *M. varia* | Anaga | Anaga | 11 | 28,54328 | -16,20538 | 7 | Meimberg 26 |
| *M. varia* | Anaga | Anaga | 12 | 28,54757 | -16,21141 | 5 | Meimberg 27 |
| *M. varia* | Anaga | Anaga | 13 | 28,54597 | -16,21688 | 7 | Meimberg 28 |
| *M. varia* | Anaga | Anaga | 14 | 28,53219 | -16,25843 | 7 | Meimberg 29 |
| *M. varia* | Anaga | S. Andrés, Anaga | 15 | 28,5162 | -16,1748 | 1 | Puppo 184 (TFC) |
| *M. varia* | Anaga | Bco Tahodio, Anaga | 16 | 28,5040 | -16,2644 | 3 | Puppo 193-195 (TFC) |
| *M. varia* | Anaga | Parque Rural, Anaga | 17 | 28,5296 | -16,1941 | 2 | Puppo 196, 197 (TFC) |
| *M. varia* | Anaga | Parque Rural, Anaga | 18 | 28,5626 | -16,2094 | 2 | Puppo 198, 199 (TFC) |
| *M. varia* | Anaga | Afur, Anaga | 19 | 28,5526 | -16,2378 | 2 | Puppo 204, 206 (TFC) |
| *M. varia* | Anaga | Antequera, Anaga | 20 | 28,5425 | -16,1305 | 1 | Puppo 223 (TFC) |
| *M. varia* | Anaga | Antequera, Anaga | 21 | 28,5482 | -16,1486 | 5 | Puppo 230, 232, 236, 237-238 (TFC) |
| *M. varia* | Anaga | Antequera, Anaga | 22 | 28,5434 | -16,1379 | 1 | Puppo 224 (TFC) |
| *M. varia* | Teno | Teno | 23 | 28,32792 | -16,85619 | 10 | Meimberg 31 |
| *M. varia* | Teno | Teno | 24 | 28,37647 | -16,85258 | 5 | Meimberg 32 |
| *M. varia* | Teno | Teno | 25 | 28,3421 | -16,8615 | 6 | Puppo 247-248, 250-253 (TFC) |
| *M. varia* | Teno | Bujame, Teno | 26 | 28,3518 | -16,8717 | 2 | Puppo 261-262 (TFC) |
| *M. lachnophylla* | Teide | El Portillo | 27 | 28,3091 | -16,5672 | 3 | Puppo 290, 294, 296 (TFC) |
| *M. lachnophylla* | Teide | ca. El Portillo | 28 | 28,31343 | -16,57074 | 2 | Meimberg 12 |
| *M. lachnophylla* | Teide | ca. El Portillo | 29 | 28,31593 | -16,5743 | 2 | Meimberg 13 |
| *M. lachnophylla* | Teide | ca. El Portillo | 30 | 28,30353 | -16,56701 | 12 | Meimberg 11 |
| *M. hyssopifolia var. glabrescens* | Northen Coast | Guancha | 31 | 28,38088889 | -16,65402778 | 2 | Puppo 561 (TFC) |
| *M. hyssopifolia var. glabrescens* | Northen Coast | Guancha | 32 | 28,36280556 | -16,65727778 | 2 | Puppo 589 (TFC) |
| *M. hyssopifolia var. glabrescens* | Northen Coast | Guancha | 33 | 28,4000 | -16,6647 | 1 | Puppo 563 (TFC) |
| *M. hyssopifolia var. glabrescens* | Northen Coast | Mirador de Mazapé | 35 | 28,3895 | -16,63627778 | 2 | Puppo 588 (TFC) |
| *M. hyssopifolia var. glabrescens* | Northen Coast | Rambla de Castro | 36 | 28,39525 | -16,62472222 | 1 | Puppo 560 (TFC) |
| *M. hyssopifolia var. glabrescens* | Northen Coast | Rambla de Castro | 37 | 28,39592 | -16,58981 | 3 | Puppo 239, 241, 243 (TFC) |
| *M. hyssopifolia var. glabrescens* | Northen Coast | Realejos | 38 | 28,37808333 | -16,6295 | 2 | Puppo 587 (TFC) |
| *M. hyssopifolia var. kuegleri* | Southern Coast | Los Abades | 39 | 28,14114 | -16,45456 | 3 | Puppo 175-176, 178 (TFC) |
| *M. hyssopifolia var. kuegleri* | Southern Coast | Acantilado de la Hondura | 40 | 28,200861 | -16,424861 | 3 | Puppo 179-181 (TFC) |
| *M. hyssopifolia var. hyssopifolia* | Southern Coast | Arico | 42 | 28,18306 | -16,45317 | 3 | Puppo 169-171 (TFC) |
| *M. hyssopifolia var. hyssopifolia* | Southern Coast | Arico | 43 | 28,15231 | -16,49158 | 1 | Puppo 172 (TFC) |
| *M. hyssopifolia var. hyssopifolia* | Southern Coast | Btw Fasnia y Güímar | 44 | 28,2497 | -16,4382 | 2 | Puppo 162, 165 (TFC) |
| *M. hyssopifolia var. hyssopifolia* | Southern Coast | East coast | 45 | 28,304 | -16,38094 | 6 | Meimberg 7 |
| *M. hyssopifolia var. hyssopifolia* | Southern Coast | Güímar | 46 | 28,2944 | -16,403 | 1 | Puppo 153 (TFC) |
| *M. hyssopifolia var. hyssopifolia* | Southern Coast | Southeast, nr Fasnia | 59 | 28,24405 | -16,40731 | 5 | Meimberg 6 |
| *M. hyssopifolia var. hyssopifolia* | Southern Coast | Southeast, nr Fasnia | 60 | 28,20048 | -16,42502 | 6 | Meimberg 8 |
| *M. hyssopifolia var. hyssopifolia* | Northeast | Arafo | 41 | 28,37269 | -16,42764 | 3 | Puppo 268-269, 272 (TFC) |
| *M. hyssopifolia var. hyssopifolia* | Northeast | Northeast | 47 | 28,33034 | -16,53259 | 5 | Meimberg 14 |
| *M. hyssopifolia var. hyssopifolia* | Northeast | Northeast | 48 | 28,34888 | -16,52472 | 9 | Meimberg 15 |
| *M. hyssopifolia var. hyssopifolia* | Northeast | Northeast | 49 | 28,3614 | -16,50061 | 4 | Meimberg 16 |
| *M. hyssopifolia var. hyssopifolia* | North | Northwest | 50 | 28,23585 | -16,7598 | 6 | Meimberg 3, 10 |
| *M. hyssopifolia var. hyssopifolia* | North | West coast | 66 | 28,15732 | -16,79511 | 3 | Meimberg 18 |
| *M. hyssopifolia var. hyssopifolia* | Adeje | South, nr Adeje | 51 | 28,06075 | -16,66895 | 8 | Meimberg 20 |
| *M. hyssopifolia var. hyssopifolia* | Adeje | Adeje | 52 | 28,14551 | -16,74021 | 11 | Meimberg 19 |
| *M. hyssopifolia var. hyssopifolia* | South | Southcenter | 53 | 28,17351 | -16,65225 | 3 | Meimberg 4 |
| *M. hyssopifolia var. hyssopifolia* | South | Southcenter | 54 | 28,14278 | -16,65101 | 3 | Meimberg 5 |
| *M. hyssopifolia var. hyssopifolia* | Southeast | Southeast | 55 | 28,20875 | -16,5392 | 10 | Meimberg 22 |
| *M. hyssopifolia var. hyssopifolia* | Southeast | Southeast | 56 | 28,20578 | -16,53791 | 3 | Meimberg 23 |
| *M. hyssopifolia var. hyssopifolia* | Southeast | Southeast | 57 | 28,19777 | -16,53239 | 2 | Meimberg 24 |
| *M. hyssopifolia var. hyssopifolia* | Southeast | Southeast | 58 | 28,16728 | -16,50658 | 9 | Meimberg 21 |
| *M. hyssopifolia var. hyssopifolia* | Southeast | Southeast | 65 | 28,23511 | -16,48109 | 5 | Meimberg 9 |
| *M. hyssopifolia var. glabrescens* | Teno | Lomo Morin, Teno | 34 | 28,35981 | -16,78911 | 2 | Puppo 562 (TFC) |
| *M. hyssopifolia var. hyssopifolia* | Teno | Teno | 61 | 28,30052 | -16,82512 | 10 | Meimberg 30 |
| *M. hyssopifolia var. hyssopifolia* | Teno | Teno | 62 | 28,26191 | -16,82088 | 3 | Meimberg 1 |
| *M. hyssopifolia var. hyssopifolia* | Teno | Teno | 63 | 28,28123 | -16,81356 | 5 | Meimberg 17 |
| *M. hyssopifolia var. hyssopifolia* | Teno | nr Teno | 64 | 28,2516 | -16,81083 | 4 | Meimberg 2 |
| Total |  |  |  |  |  | 289 |  |

* No geographical coordinates are provided for restricted species; locality numbers correspond to those indicated in Fig. 1.
