# Supplementary material for: Genetic structure of Micromeria (Lamiaceae) in Tenerife, the imprint of geological history and hybridization on within‐island diversification
Source: Ecol Evol. 2016 Apr 20;6(11):3443–60. doi: 10.1002/ece3.2094 (PMC5513284; doi:10.1002/ece3.2094)
Supplement: Supplementary file 3 — Table S2. Results for HWE and Bottleneck test per population. Here, we present the number of loci deviating from HWE and the P‐value for deviations from the mutation‐drift equilibrium (Bottleneck). [file ECE3-6-3443-s003.docx]

**Table S2** Results for HWE and Bottleneck test per population. Here we present the number of loci deviating from HWE and the p-value for deviations the from mutation-drift equilibrium (Bottleneck).

| **Population** | **Species** | **Region** | **nr. Loci deviating from HWE** | **Bottleneck p-value** |
| --- | --- | --- | --- | --- |
| *3* | *M. teneriffae* | Anaga | 1 | 0.330 |
| *5* | *M. teneriffae* | Southern Coast | 1 | 0.593 |
| *7* | *M. glomerata* | Anaga | 3 | 0.039 |
| *8* | *M. rivas-martninezii* | Anaga | 5 | 0.219 |
| *9* | *M. densiflora* | Teno | 0 | 0.031 |
| *10* | *M. lasiophylla* | Teide | 0 | 0.352 |
| *11* | *M. varia* | Anaga | 2 | 0.389 |
| *12* | *M. varia* | Anaga | 5 | 0.580 |
| *13* | *M. varia* | Anaga | 0 | 0.365 |
| *14* | *M. varia* | Anaga | 2 | 0.572 |
| *21* | *M. varia* | Anaga | 0 | 0.068 |
| *23* | *M. varia* | Teno | 1 | 0.195 |
| *24* | *M. varia* | Teno | 0 | 0.014 |
| *25* | *M. varia* | Teno | 1 | 0.190 |
| *30* | *M. lachnophylla* | Teide | 3 | 0.225 |
| *45* | *M. hyssopifolia* | Southern Coast | 0 | 0.439 |
| *47* | *M. hyssopifolia* | North east | 2 | 0.461 |
| *48* | *M. hyssopifolia* | North east | 2 | 0.109 |
| *50* | *M. hyssopifolia* | North west | 2 | 0.499 |
| *51* | *M. hyssopifolia* | Adeje | 2 | 0.407 |
| *52* | *M. hyssopifolia* | Adeje | 5 | 0.573 |
| *55* | *M. hyssopifolia* | Southeast | 4 | 0.573 |
| *58* | *M. hyssopifolia* | Southeast | 3 | 0.039 |
| *59* | *M. hyssopifolia* | Southern Coast | 2 | 0.377 |
| *60* | *M. hyssopifolia* | Southern Coast | 1 | 0.318 |
| *61* | *M. hyssopifolia* | Teno | 3 | 0.390 |
| *63* | *M. hyssopifolia* | Teno | 4 | 0.532 |
| *65* | *M. hyssopifolia* | Southeast | 3 | 0.144 |
