# Supplementary material for: Genetic structure of Micromeria (Lamiaceae) in Tenerife, the imprint of geological history and hybridization on within‐island diversification
Source: Ecol Evol. 2016 Apr 20;6(11):3443–60. doi: 10.1002/ece3.2094 (PMC5513284; doi:10.1002/ece3.2094)
Supplement: Supplementary file 4 — Table S3. List of pairwise Fst and unbiased Nei distance results for all populations with at least four individuals. [file ECE3-6-3443-s004.docx]

**Table S3** List of pairwise Fst and unbiased Nei distance results for all populations with at least four individuals.

| **Pop1** | **Region 1** | **Species 1** | **Pop2** | **Region** | **Species** | **Nei uD** | **Fst** |
| --- | --- | --- | --- | --- | --- | --- | --- |
| 1 | Southern coast | *M. teneriffae* | 5 | Southern coast | *M. teneriffae* | 0.499 | 0.118 |
| 1 | Southern coast | *M. teneriffae* | 11 | Anaga | *M. varia* | 0.891 | 0.123 |
| 1 | Southern coast | *M. teneriffae* | 12 | Anaga | *M. varia* | 1.050 | 0.228 |
| 1 | Southern coast | *M. teneriffae* | 13 | Anaga | *M. varia* | 0.991 | 0.196 |
| 1 | Southern coast | *M. teneriffae* | 14 | Anaga | *M. varia* | 0.883 | 0.223 |
| 1 | Southern coast | *M. teneriffae* | 21 | Anaga | *M. varia* | 0.628 | 0.127 |
| 1 | Southern coast | *M. teneriffae* | 23 | Teno | *M. varia* | 1.033 | 0.244 |
| 1 | Southern coast | *M. teneriffae* | 24 | Teno | *M. varia* | 1.248 | 0.147 |
| 1 | Southern coast | *M. teneriffae* | 25 | Teno | *M. varia* | 1.146 | 0.175 |
| 3 | Anaga | *M. teneriffae* | 1 | Southern coast | *M. teneriffae* | 0.500 | 0.133 |
| 3 | Anaga | *M. teneriffae* | 5 | Southern coast | *M. teneriffae* | 0.129 | 0.090 |
| 3 | Anaga | *M. teneriffae* | 11 | Anaga | *M. varia* | 0.364 | 0.102 |
| 3 | Anaga | *M. teneriffae* | 12 | Anaga | *M. varia* | 0.331 | 0.101 |
| 3 | Anaga | *M. teneriffae* | 13 | Anaga | *M. varia* | 0.298 | 0.111 |
| 3 | Anaga | *M. teneriffae* | 14 | Anaga | *M. varia* | 0.466 | 0.110 |
| 3 | Anaga | *M. teneriffae* | 21 | Anaga | *M. varia* | 0.160 | 0.099 |
| 3 | Anaga | *M. teneriffae* | 23 | Teno | *M. varia* | 0.242 | 0.120 |
| 3 | Anaga | *M. teneriffae* | 24 | Teno | *M. varia* | 0.441 | 0.091 |
| 3 | Anaga | *M. teneriffae* | 25 | Teno | *M. varia* | 0.371 | 0.134 |
| 5 | Southern coast | *M. teneriffae* | 11 | Anaga | *M. varia* | 0.708 | 0.169 |
| 5 | Southern coast | *M. teneriffae* | 12 | Anaga | *M. varia* | 0.560 | 0.169 |
| 5 | Southern coast | *M. teneriffae* | 13 | Anaga | *M. varia* | 0.607 | 0.165 |
| 5 | Southern coast | *M. teneriffae* | 14 | Anaga | *M. varia* | 0.507 | 0.150 |
| 5 | Southern coast | *M. teneriffae* | 21 | Anaga | *M. varia* | 0.388 | 0.095 |
| 5 | Southern coast | *M. teneriffae* | 23 | Teno | *M. varia* | 0.450 | 0.259 |
| 5 | Southern coast | *M. teneriffae* | 24 | Teno | *M. varia* | 0.571 | 0.282 |
| 5 | Southern coast | *M. teneriffae* | 25 | Teno | *M. varia* | 0.442 | 0.188 |
| 7 | Anaga | *M. glomerata* | 1 | Southern coast | *M. teneriffae* | 1.635 | 0.104 |
| 7 | Anaga | *M. glomerata* | 3 | Anaga | *M. teneriffae* | 1.259 | 0.071 |
| 7 | Anaga | *M. glomerata* | 5 | Southern coast | *M. teneriffae* | 1.272 | 0.089 |
| 7 | Anaga | *M. glomerata* | 8 | Anaga | *M. rivas-martinezii* | 0.993 | 0.122 |
| 7 | Anaga | *M. glomerata* | 10 | Teide | *M. lasiophylla* | 1.476 | 0.144 |
| 7 | Anaga | *M. glomerata* | 10 | Teide | *M. lasiophylla* | 1.505 | 0.072 |
| 7 | Anaga | *M. glomerata* | 11 | Anaga | *M. varia* | 1.676 | 0.086 |
| 7 | Anaga | *M. glomerata* | 12 | Anaga | *M. varia* | 1.587 | 0.073 |
| 7 | Anaga | *M. glomerata* | 13 | Anaga | *M. varia* | 1.702 | 0.243 |
| 7 | Anaga | *M. glomerata* | 14 | Anaga | *M. varia* | 2.177 | 0.306 |
| 7 | Anaga | *M. glomerata* | 21 | Anaga | *M. varia* | 1.051 | 0.119 |
| 7 | Anaga | *M. glomerata* | 23 | Teno | *M. varia* | 1.584 | 0.052 |
| 7 | Anaga | *M. glomerata* | 24 | Teno | *M. varia* | 1.609 | 0.102 |
| 7 | Anaga | *M. glomerata* | 25 | Teno | *M. varia* | 0.973 | 0.051 |
| 7 | Anaga | *M. glomerata* | 30 | Teide | *M. lachnophylla* | 1.385 | 0.316 |
| 7 | Anaga | *M. glomerata* | 45 | Southern coast | *M. hyssopifolia* | 1.229 | 0.310 |
| 7 | Anaga | *M. glomerata* | 47 | Northeast | *M. hyssopifolia* | 1.508 | 0.062 |
| 7 | Anaga | *M. glomerata* | 48 | Northeast | *M. hyssopifolia* | 1.507 | 0.076 |
| 7 | Anaga | *M. glomerata* | 49 | Northeast | *M. hyssopifolia* | 1.522 | 0.086 |
| 7 | Anaga | *M. glomerata* | 50 | Northeast | *M. hyssopifolia* | 1.960 | 0.237 |
| 7 | Anaga | *M. glomerata* | 51 | Adeje | *M. hyssopifolia* | 2.045 | 0.078 |
| 7 | Anaga | *M. glomerata* | 52 | Adeje | *M. hyssopifolia* | 1.625 | 0.106 |
| 7 | Anaga | *M. glomerata* | 55 | Southeast | *M. hyssopifolia* | 1.485 | 0.108 |
| 7 | Anaga | *M. glomerata* | 58 | Southeast | *M. hyssopifolia* | 1.436 | 0.060 |
| 7 | Anaga | *M. glomerata* | 59 | Southern coast | *M. hyssopifolia* | 1.578 | 0.133 |
| 7 | Anaga | *M. glomerata* | 60 | Southern coast | *M. hyssopifolia* | 1.760 | 0.050 |
| 7 | Anaga | *M. glomerata* | 61 | Teno | *M. hyssopifolia* | 1.529 | 0.065 |
| 7 | Anaga | *M. glomerata* | 63 | Teno | *M. hyssopifolia* | 1.758 | 0.069 |
| 7 | Anaga | *M. glomerata* | 64 | Teno | *M. hyssopifolia* | 1.234 | 0.262 |
| 7 | Anaga | *M. glomerata* | 65 | Southeast | *M. hyssopifolia* | 1.657 | 0.099 |
| 8 | Anaga | *M. rivas-martinezii* | 1 | Southern coast | *M. teneriffae* | 0.890 | 0.091 |
| 8 | Anaga | *M. rivas-martinezii* | 3 | Anaga | *M. teneriffae* | 0.420 | 0.099 |
| 8 | Anaga | *M. rivas-martinezii* | 5 | Southern coast | *M. teneriffae* | 0.348 | 0.098 |
| 8 | Anaga | *M. rivas-martinezii* | 11 | Anaga | *M. varia* | 0.902 | 0.099 |
| 8 | Anaga | *M. rivas-martinezii* | 12 | Anaga | *M. varia* | 0.607 | 0.094 |
| 8 | Anaga | *M. rivas-martinezii* | 13 | Anaga | *M. varia* | 0.699 | 0.286 |
| 8 | Anaga | *M. rivas-martinezii* | 14 | Anaga | *M. varia* | 0.746 | 0.360 |
| 8 | Anaga | *M. rivas-martinezii* | 21 | Anaga | *M. varia* | 0.438 | 0.161 |
| 8 | Anaga | *M. rivas-martinezii* | 23 | Teno | *M. varia* | 0.761 | 0.114 |
| 8 | Anaga | *M. rivas-martinezii* | 24 | Teno | *M. varia* | 0.980 | 0.157 |
| 8 | Anaga | *M. rivas-martinezii* | 25 | Teno | *M. varia* | 0.784 | 0.124 |
| 9 | Teno | *M. densiflora* | 1 | Southern coast | *M. teneriffae* | 1.489 | 0.266 |
| 9 | Teno | *M. densiflora* | 3 | Anaga | *M. teneriffae* | 1.001 | 0.065 |
| 9 | Teno | *M. densiflora* | 5 | Southern coast | *M. teneriffae* | 0.783 | 0.343 |
| 9 | Teno | *M. densiflora* | 7 | Anaga | *M. glomerata* | 1.645 | 0.500 |
| 9 | Teno | *M. densiflora* | 8 | Anaga | *M. rivas-martinezii* | 0.874 | 0.112 |
| 9 | Teno | *M. densiflora* | 10 | Teide | *M. lasiophylla* | 1.022 | 0.151 |
| 9 | Teno | *M. densiflora* | 10 | Teide | *M. lasiophylla* | 1.012 | 0.099 |
| 9 | Teno | *M. densiflora* | 11 | Anaga | *M. varia* | 1.337 | 0.123 |
| 9 | Teno | *M. densiflora* | 12 | Anaga | *M. varia* | 0.832 | 0.071 |
| 9 | Teno | *M. densiflora* | 13 | Anaga | *M. varia* | 1.341 | 0.108 |
| 9 | Teno | *M. densiflora* | 14 | Anaga | *M. varia* | 0.889 | 0.067 |
| 9 | Teno | *M. densiflora* | 21 | Anaga | *M. varia* | 1.128 | 0.097 |
| 9 | Teno | *M. densiflora* | 23 | Teno | *M. varia* | 0.770 | 0.210 |
| 9 | Teno | *M. densiflora* | 24 | Teno | *M. varia* | 0.808 | 0.295 |
| 9 | Teno | *M. densiflora* | 25 | Teno | *M. varia* | 0.713 | 0.153 |
| 9 | Teno | *M. densiflora* | 30 | Teide | *M. lachnophylla* | 0.869 | 0.333 |
| 9 | Teno | *M. densiflora* | 45 | Southern coast | *M. hyssopifolia* | 0.517 | 0.345 |
| 9 | Teno | *M. densiflora* | 47 | Northeast | *M. hyssopifolia* | 0.766 | 0.268 |
| 9 | Teno | *M. densiflora* | 48 | Northeast | *M. hyssopifolia* | 0.748 | 0.309 |
| 9 | Teno | *M. densiflora* | 49 | Northeast | *M. hyssopifolia* | 1.143 | 0.092 |
| 9 | Teno | *M. densiflora* | 50 | Northeast | *M. hyssopifolia* | 0.892 | 0.278 |
| 9 | Teno | *M. densiflora* | 51 | Adeje | *M. hyssopifolia* | 1.553 | 0.389 |
| 9 | Teno | *M. densiflora* | 52 | Adeje | *M. hyssopifolia* | 1.081 | 0.412 |
| 9 | Teno | *M. densiflora* | 55 | Southeast | *M. hyssopifolia* | 0.808 | 0.233 |
| 9 | Teno | *M. densiflora* | 58 | Southeast | *M. hyssopifolia* | 0.754 | 0.299 |
| 9 | Teno | *M. densiflora* | 59 | Southern coast | *M. hyssopifolia* | 0.667 | 0.192 |
| 9 | Teno | *M. densiflora* | 60 | Southern coast | *M. hyssopifolia* | 0.895 | 0.106 |
| 9 | Teno | *M. densiflora* | 61 | Teno | *M. hyssopifolia* | 0.817 | 0.060 |
| 9 | Teno | *M. densiflora* | 63 | Teno | *M. hyssopifolia* | 0.985 | 0.081 |
| 9 | Teno | *M. densiflora* | 64 | Teno | *M. hyssopifolia* | 0.964 | 0.300 |
| 9 | Teno | *M. densiflora* | 65 | Southeast | *M. hyssopifolia* | 0.788 | 0.135 |
| 10 | Teide | *M. lasiophylla* | 1 | Southern coast | *M. teneriffae* | 0.664 | 0.138 |
| 10 | Teide | *M. lasiophylla* | 1 | Southern coast | *M. teneriffae* | 0.786 | 0.042 |
| 10 | Teide | *M. lasiophylla* | 3 | Anaga | *M. teneriffae* | 0.533 | 0.311 |
| 10 | Teide | *M. lasiophylla* | 3 | Anaga | *M. teneriffae* | 0.672 | 0.069 |
| 10 | Teide | *M. lasiophylla* | 5 | Southern coast | *M. teneriffae* | 0.468 | 0.058 |
| 10 | Teide | *M. lasiophylla* | 5 | Southern coast | *M. teneriffae* | 0.577 | 0.094 |
| 10 | Teide | *M. lasiophylla* | 8 | Anaga | *M. rivas-martinezii* | 0.541 | 0.250 |
| 10 | Teide | *M. lasiophylla* | 8 | Anaga | *M. rivas-martinezii* | 0.615 | 0.070 |
| 10 | Teide | *M. lasiophylla* | 10 | Teide | *M. lasiophylla* | 0.275 | 0.104 |
| 10 | Teide | *M. lasiophylla* | 11 | Anaga | *M. varia* | 0.922 | 0.099 |
| 10 | Teide | *M. lasiophylla* | 11 | Anaga | *M. varia* | 1.115 | 0.086 |
| 10 | Teide | *M. lasiophylla* | 12 | Anaga | *M. varia* | 0.776 | 0.061 |
| 10 | Teide | *M. lasiophylla* | 12 | Anaga | *M. varia* | 0.797 | 0.062 |
| 10 | Teide | *M. lasiophylla* | 13 | Anaga | *M. varia* | 0.942 | 0.092 |
| 10 | Teide | *M. lasiophylla* | 13 | Anaga | *M. varia* | 1.136 | 0.169 |
| 10 | Teide | *M. lasiophylla* | 14 | Anaga | *M. varia* | 0.833 | 0.067 |
| 10 | Teide | *M. lasiophylla* | 14 | Anaga | *M. varia* | 1.016 | 0.150 |
| 10 | Teide | *M. lasiophylla* | 21 | Anaga | *M. varia* | 0.495 | 0.077 |
| 10 | Teide | *M. lasiophylla* | 21 | Anaga | *M. varia* | 0.644 | 0.165 |
| 10 | Teide | *M. lasiophylla* | 23 | Teno | *M. varia* | 0.720 | 0.055 |
| 10 | Teide | *M. lasiophylla* | 23 | Teno | *M. varia* | 0.661 | 0.076 |
| 10 | Teide | *M. lasiophylla* | 24 | Teno | *M. varia* | 1.083 | 0.077 |
| 10 | Teide | *M. lasiophylla* | 24 | Teno | *M. varia* | 0.824 | 0.197 |
| 10 | Teide | *M. lasiophylla* | 25 | Teno | *M. varia* | 0.751 | 0.050 |
| 10 | Teide | *M. lasiophylla* | 25 | Teno | *M. varia* | 0.711 | 0.103 |
| 11 | Anaga | *M. varia* | 12 | Anaga | *M. varia* | 0.129 | 0.102 |
| 11 | Anaga | *M. varia* | 13 | Anaga | *M. varia* | 0.130 | 0.139 |
| 11 | Anaga | *M. varia* | 14 | Anaga | *M. varia* | 0.370 | 0.098 |
| 11 | Anaga | *M. varia* | 21 | Anaga | *M. varia* | 0.135 | 0.126 |
| 11 | Anaga | *M. varia* | 23 | Teno | *M. varia* | 0.420 | 0.096 |
| 11 | Anaga | *M. varia* | 24 | Teno | *M. varia* | 0.865 | 0.098 |
| 11 | Anaga | *M. varia* | 25 | Teno | *M. varia* | 0.546 | 0.084 |
| 12 | Anaga | *M. varia* | 13 | Anaga | *M. varia* | 0.142 | 0.114 |
| 12 | Anaga | *M. varia* | 14 | Anaga | *M. varia* | 0.253 | 0.076 |
| 12 | Anaga | *M. varia* | 21 | Anaga | *M. varia* | 0.125 | 0.099 |
| 12 | Anaga | *M. varia* | 23 | Teno | *M. varia* | 0.310 | 0.100 |
| 12 | Anaga | *M. varia* | 24 | Teno | *M. varia* | 0.755 | 0.069 |
| 12 | Anaga | *M. varia* | 25 | Teno | *M. varia* | 0.415 | 0.120 |
| 13 | Anaga | *M. varia* | 14 | Anaga | *M. varia* | 0.273 | 0.114 |
| 13 | Anaga | *M. varia* | 21 | Anaga | *M. varia* | 0.225 | 0.078 |
| 13 | Anaga | *M. varia* | 23 | Teno | *M. varia* | 0.361 | 0.182 |
| 13 | Anaga | *M. varia* | 24 | Teno | *M. varia* | 0.655 | 0.168 |
| 13 | Anaga | *M. varia* | 25 | Teno | *M. varia* | 0.509 | 0.183 |
| 14 | Anaga | *M. varia* | 21 | Anaga | *M. varia* | 0.346 | 0.108 |
| 14 | Anaga | *M. varia* | 23 | Teno | *M. varia* | 0.424 | 0.220 |
| 14 | Anaga | *M. varia* | 24 | Teno | *M. varia* | 0.786 | 0.119 |
| 14 | Anaga | *M. varia* | 25 | Teno | *M. varia* | 0.654 | 0.133 |
| 21 | Anaga | *M. varia* | 23 | Teno | *M. varia* | 0.354 | 0.121 |
| 21 | Anaga | *M. varia* | 24 | Teno | *M. varia* | 0.612 | 0.135 |
| 21 | Anaga | *M. varia* | 25 | Teno | *M. varia* | 0.459 | 0.137 |
| 23 | Teno | *M. varia* | 24 | Teno | *M. varia* | 0.293 | 0.121 |
| 23 | Teno | *M. varia* | 25 | Teno | *M. varia* | 0.098 | 0.060 |
| 24 | Teno | *M. varia* | 25 | Teno | *M. varia* | 0.402 | 0.129 |
| 30 | Teide | *M. lachnophylla* | 1 | Southern coast | *M. teneriffae* | 0.609 | 0.100 |
| 30 | Teide | *M. lachnophylla* | 3 | Anaga | *M. teneriffae* | 0.380 | 0.128 |
| 30 | Teide | *M. lachnophylla* | 5 | Southern coast | *M. teneriffae* | 0.316 | 0.150 |
| 30 | Teide | *M. lachnophylla* | 8 | Anaga | *M. rivas-martinezii* | 0.654 | 0.146 |
| 30 | Teide | *M. lachnophylla* | 10 | Teide | *M. lasiophylla* | 0.540 | 0.125 |
| 30 | Teide | *M. lachnophylla* | 10 | Teide | *M. lasiophylla* | 0.545 | 0.096 |
| 30 | Teide | *M. lachnophylla* | 11 | Anaga | *M. varia* | 0.674 | 0.159 |
| 30 | Teide | *M. lachnophylla* | 12 | Anaga | *M. varia* | 0.448 | 0.139 |
| 30 | Teide | *M. lachnophylla* | 13 | Anaga | *M. varia* | 0.503 | 0.075 |
| 30 | Teide | *M. lachnophylla* | 14 | Anaga | *M. varia* | 0.617 | 0.171 |
| 30 | Teide | *M. lachnophylla* | 21 | Anaga | *M. varia* | 0.440 | 0.112 |
| 30 | Teide | *M. lachnophylla* | 23 | Teno | *M. varia* | 0.254 | 0.074 |
| 30 | Teide | *M. lachnophylla* | 24 | Teno | *M. varia* | 0.552 | 0.073 |
| 30 | Teide | *M. lachnophylla* | 25 | Teno | *M. varia* | 0.258 | 0.092 |
| 45 | Southern coast | *M. hyssopifolia* | 1 | Southern coast | *M. teneriffae* | 0.820 | 0.257 |
| 45 | Southern coast | *M. hyssopifolia* | 3 | Anaga | *M. teneriffae* | 0.374 | 0.392 |
| 45 | Southern coast | *M. hyssopifolia* | 5 | Southern coast | *M. teneriffae* | 0.291 | 0.164 |
| 45 | Southern coast | *M. hyssopifolia* | 8 | Anaga | *M. rivas-martinezii* | 0.515 | 0.396 |
| 45 | Southern coast | *M. hyssopifolia* | 10 | Teide | *M. lasiophylla* | 0.540 | 0.164 |
| 45 | Southern coast | *M. hyssopifolia* | 10 | Teide | *M. lasiophylla* | 0.728 | 0.132 |
| 45 | Southern coast | *M. hyssopifolia* | 11 | Anaga | *M. varia* | 0.431 | 0.189 |
| 45 | Southern coast | *M. hyssopifolia* | 12 | Anaga | *M. varia* | 0.418 | 0.141 |
| 45 | Southern coast | *M. hyssopifolia* | 13 | Anaga | *M. varia* | 0.495 | 0.187 |
| 45 | Southern coast | *M. hyssopifolia* | 14 | Anaga | *M. varia* | 0.303 | 0.202 |
| 45 | Southern coast | *M. hyssopifolia* | 21 | Anaga | *M. varia* | 0.386 | 0.178 |
| 45 | Southern coast | *M. hyssopifolia* | 23 | Teno | *M. varia* | 0.295 | 0.165 |
| 45 | Southern coast | *M. hyssopifolia* | 24 | Teno | *M. varia* | 0.283 | 0.175 |
| 45 | Southern coast | *M. hyssopifolia* | 25 | Teno | *M. varia* | 0.330 | 0.184 |
| 45 | Southern coast | *M. hyssopifolia* | 30 | Teide | *M. lachnophylla* | 0.383 | 0.151 |
| 45 | Southern coast | *M. hyssopifolia* | 55 | Southeast | *M. hyssopifolia* | 0.190 | 0.089 |
| 45 | Southern coast | *M. hyssopifolia* | 58 | Southeast | *M. hyssopifolia* | 0.158 | 0.094 |
| 45 | Southern coast | *M. hyssopifolia* | 59 | Southern coast | *M. hyssopifolia* | 0.165 | 0.082 |
| 45 | Southern coast | *M. hyssopifolia* | 60 | Southern coast | *M. hyssopifolia* | 0.339 | 0.074 |
| 45 | Southern coast | *M. hyssopifolia* | 61 | Teno | *M. hyssopifolia* | 0.160 | 0.127 |
| 45 | Southern coast | *M. hyssopifolia* | 63 | Teno | *M. hyssopifolia* | 0.419 | 0.104 |
| 45 | Southern coast | *M. hyssopifolia* | 64 | Teno | *M. hyssopifolia* | 0.226 | 0.090 |
| 45 | Southern coast | *M. hyssopifolia* | 65 | Southeast | *M. hyssopifolia* | 0.200 | 0.083 |
| 47 | Northeast | *M. hyssopifolia* | 1 | Southern coast | *M. teneriffae* | 0.725 | 0.110 |
| 47 | Northeast | *M. hyssopifolia* | 3 | Anaga | *M. teneriffae* | 0.420 | 0.059 |
| 47 | Northeast | *M. hyssopifolia* | 5 | Southern coast | *M. teneriffae* | 0.344 | 0.250 |
| 47 | Northeast | *M. hyssopifolia* | 8 | Anaga | *M. rivas-martinezii* | 0.780 | 0.096 |
| 47 | Northeast | *M. hyssopifolia* | 10 | Teide | *M. lasiophylla* | 0.564 | 0.067 |
| 47 | Northeast | *M. hyssopifolia* | 10 | Teide | *M. lasiophylla* | 0.484 | 0.074 |
| 47 | Northeast | *M. hyssopifolia* | 11 | Anaga | *M. varia* | 0.619 | 0.295 |
| 47 | Northeast | *M. hyssopifolia* | 12 | Anaga | *M. varia* | 0.508 | 0.138 |
| 47 | Northeast | *M. hyssopifolia* | 13 | Anaga | *M. varia* | 0.644 | 0.069 |
| 47 | Northeast | *M. hyssopifolia* | 14 | Anaga | *M. varia* | 0.540 | 0.105 |
| 47 | Northeast | *M. hyssopifolia* | 21 | Anaga | *M. varia* | 0.469 | 0.061 |
| 47 | Northeast | *M. hyssopifolia* | 23 | Teno | *M. varia* | 0.349 | 0.102 |
| 47 | Northeast | *M. hyssopifolia* | 24 | Teno | *M. varia* | 0.589 | 0.081 |
| 47 | Northeast | *M. hyssopifolia* | 25 | Teno | *M. varia* | 0.483 | 0.083 |
| 47 | Northeast | *M. hyssopifolia* | 30 | Teide | *M. lachnophylla* | 0.402 | 0.091 |
| 47 | Northeast | *M. hyssopifolia* | 45 | Southern coast | *M. hyssopifolia* | 0.358 | 0.322 |
| 47 | Northeast | *M. hyssopifolia* | 48 | Northeast | *M. hyssopifolia* | 0.211 | 0.095 |
| 47 | Northeast | *M. hyssopifolia* | 49 | Northeast | *M. hyssopifolia* | 0.270 | 0.085 |
| 47 | Northeast | *M. hyssopifolia* | 50 | Northeast | *M. hyssopifolia* | 0.360 | 0.301 |
| 47 | Northeast | *M. hyssopifolia* | 55 | Southeast | *M. hyssopifolia* | 0.388 | 0.150 |
| 47 | Northeast | *M. hyssopifolia* | 58 | Southeast | *M. hyssopifolia* | 0.350 | 0.083 |
| 47 | Northeast | *M. hyssopifolia* | 59 | Southern coast | *M. hyssopifolia* | 0.364 | 0.116 |
| 47 | Northeast | *M. hyssopifolia* | 60 | Southern coast | *M. hyssopifolia* | 0.530 | 0.063 |
| 47 | Northeast | *M. hyssopifolia* | 61 | Teno | *M. hyssopifolia* | 0.341 | 0.066 |
| 47 | Northeast | *M. hyssopifolia* | 63 | Teno | *M. hyssopifolia* | 0.547 | 0.093 |
| 47 | Northeast | *M. hyssopifolia* | 64 | Teno | *M. hyssopifolia* | 0.591 | 0.077 |
| 47 | Northeast | *M. hyssopifolia* | 65 | Southeast | *M. hyssopifolia* | 0.223 | 0.117 |
| 48 | Northeast | *M. hyssopifolia* | 1 | Southern coast | *M. teneriffae* | 0.555 | 0.151 |
| 48 | Northeast | *M. hyssopifolia* | 3 | Anaga | *M. teneriffae* | 0.229 | 0.165 |
| 48 | Northeast | *M. hyssopifolia* | 5 | Southern coast | *M. teneriffae* | 0.274 | 0.150 |
| 48 | Northeast | *M. hyssopifolia* | 8 | Anaga | *M. rivas-martinezii* | 0.570 | 0.181 |
| 48 | Northeast | *M. hyssopifolia* | 10 | Teide | *M. lasiophylla* | 0.439 | 0.165 |
| 48 | Northeast | *M. hyssopifolia* | 10 | Teide | *M. lasiophylla* | 0.413 | 0.123 |
| 48 | Northeast | *M. hyssopifolia* | 11 | Anaga | *M. varia* | 0.322 | 0.181 |
| 48 | Northeast | *M. hyssopifolia* | 12 | Anaga | *M. varia* | 0.249 | 0.150 |
| 48 | Northeast | *M. hyssopifolia* | 13 | Anaga | *M. varia* | 0.317 | 0.144 |
| 48 | Northeast | *M. hyssopifolia* | 14 | Anaga | *M. varia* | 0.239 | 0.173 |
| 48 | Northeast | *M. hyssopifolia* | 21 | Anaga | *M. varia* | 0.272 | 0.160 |
| 48 | Northeast | *M. hyssopifolia* | 23 | Teno | *M. varia* | 0.225 | 0.207 |
| 48 | Northeast | *M. hyssopifolia* | 24 | Teno | *M. varia* | 0.561 | 0.190 |
| 48 | Northeast | *M. hyssopifolia* | 25 | Teno | *M. varia* | 0.406 | 0.138 |
| 48 | Northeast | *M. hyssopifolia* | 30 | Teide | *M. lachnophylla* | 0.255 | 0.148 |
| 48 | Northeast | *M. hyssopifolia* | 45 | Southern coast | *M. hyssopifolia* | 0.185 | 0.064 |
| 48 | Northeast | *M. hyssopifolia* | 49 | Northeast | *M. hyssopifolia* | 0.111 | 0.054 |
| 48 | Northeast | *M. hyssopifolia* | 50 | Northeast | *M. hyssopifolia* | 0.217 | 0.080 |
| 48 | Northeast | *M. hyssopifolia* | 55 | Southeast | *M. hyssopifolia* | 0.164 | 0.065 |
| 48 | Northeast | *M. hyssopifolia* | 58 | Southeast | *M. hyssopifolia* | 0.218 | 0.101 |
| 48 | Northeast | *M. hyssopifolia* | 59 | Southern coast | *M. hyssopifolia* | 0.162 | 0.064 |
| 48 | Northeast | *M. hyssopifolia* | 60 | Southern coast | *M. hyssopifolia* | 0.271 | 0.074 |
| 48 | Northeast | *M. hyssopifolia* | 61 | Teno | *M. hyssopifolia* | 0.197 | 0.334 |
| 48 | Northeast | *M. hyssopifolia* | 63 | Teno | *M. hyssopifolia* | 0.382 | 0.372 |
| 48 | Northeast | *M. hyssopifolia* | 64 | Teno | *M. hyssopifolia* | 0.236 | 0.245 |
| 48 | Northeast | *M. hyssopifolia* | 65 | Southeast | *M. hyssopifolia* | 0.169 | 0.099 |
| 49 | Northeast | *M. hyssopifolia* | 1 | Southern coast | *M. teneriffae* | 0.755 | 0.189 |
| 49 | Northeast | *M. hyssopifolia* | 3 | Anaga | *M. teneriffae* | 0.215 | 0.163 |
| 49 | Northeast | *M. hyssopifolia* | 5 | Southern coast | *M. teneriffae* | 0.368 | 0.190 |
| 49 | Northeast | *M. hyssopifolia* | 8 | Anaga | *M. rivas-martinezii* | 0.556 | 0.156 |
| 49 | Northeast | *M. hyssopifolia* | 10 | Teide | *M. lasiophylla* | 0.697 | 0.152 |
| 49 | Northeast | *M. hyssopifolia* | 10 | Teide | *M. lasiophylla* | 0.831 | 0.147 |
| 49 | Northeast | *M. hyssopifolia* | 11 | Anaga | *M. varia* | 0.204 | 0.130 |
| 49 | Northeast | *M. hyssopifolia* | 12 | Anaga | *M. varia* | 0.297 | 0.142 |
| 49 | Northeast | *M. hyssopifolia* | 13 | Anaga | *M. varia* | 0.195 | 0.299 |
| 49 | Northeast | *M. hyssopifolia* | 14 | Anaga | *M. varia* | 0.234 | 0.332 |
| 49 | Northeast | *M. hyssopifolia* | 21 | Anaga | *M. varia* | 0.327 | 0.263 |
| 49 | Northeast | *M. hyssopifolia* | 23 | Teno | *M. varia* | 0.290 | 0.153 |
| 49 | Northeast | *M. hyssopifolia* | 24 | Teno | *M. varia* | 0.417 | 0.192 |
| 49 | Northeast | *M. hyssopifolia* | 25 | Teno | *M. varia* | 0.462 | 0.138 |
| 49 | Northeast | *M. hyssopifolia* | 30 | Teide | *M. lachnophylla* | 0.469 | 0.177 |
| 49 | Northeast | *M. hyssopifolia* | 45 | Southern coast | *M. hyssopifolia* | 0.129 | 0.362 |
| 49 | Northeast | *M. hyssopifolia* | 50 | Northeast | *M. hyssopifolia* | 0.262 | 0.317 |
| 49 | Northeast | *M. hyssopifolia* | 55 | Southeast | *M. hyssopifolia* | 0.214 | 0.156 |
| 49 | Northeast | *M. hyssopifolia* | 58 | Southeast | *M. hyssopifolia* | 0.276 | 0.117 |
| 49 | Northeast | *M. hyssopifolia* | 59 | Southern coast | *M. hyssopifolia* | 0.316 | 0.248 |
| 49 | Northeast | *M. hyssopifolia* | 60 | Southern coast | *M. hyssopifolia* | 0.370 | 0.151 |
| 49 | Northeast | *M. hyssopifolia* | 61 | Teno | *M. hyssopifolia* | 0.290 | 0.153 |
| 49 | Northeast | *M. hyssopifolia* | 63 | Teno | *M. hyssopifolia* | 0.293 | 0.158 |
| 49 | Northeast | *M. hyssopifolia* | 64 | Teno | *M. hyssopifolia* | 0.360 | 0.143 |
| 49 | Northeast | *M. hyssopifolia* | 65 | Southeast | *M. hyssopifolia* | 0.247 | 0.182 |
| 50 | Northeast | *M. hyssopifolia* | 1 | Southern coast | *M. teneriffae* | 1.027 | 0.284 |
| 50 | Northeast | *M. hyssopifolia* | 3 | Anaga | *M. teneriffae* | 0.254 | 0.195 |
| 50 | Northeast | *M. hyssopifolia* | 5 | Southern coast | *M. teneriffae* | 0.359 | 0.307 |
| 50 | Northeast | *M. hyssopifolia* | 8 | Anaga | *M. rivas-martinezii* | 0.704 | 0.175 |
| 50 | Northeast | *M. hyssopifolia* | 10 | Teide | *M. lasiophylla* | 0.691 | 0.201 |
| 50 | Northeast | *M. hyssopifolia* | 10 | Teide | *M. lasiophylla* | 0.594 | 0.149 |
| 50 | Northeast | *M. hyssopifolia* | 11 | Anaga | *M. varia* | 0.579 | 0.173 |
| 50 | Northeast | *M. hyssopifolia* | 12 | Anaga | *M. varia* | 0.425 | 0.088 |
| 50 | Northeast | *M. hyssopifolia* | 13 | Anaga | *M. varia* | 0.464 | 0.117 |
| 50 | Northeast | *M. hyssopifolia* | 14 | Anaga | *M. varia* | 0.570 | 0.072 |
| 50 | Northeast | *M. hyssopifolia* | 21 | Anaga | *M. varia* | 0.451 | 0.095 |
| 50 | Northeast | *M. hyssopifolia* | 23 | Teno | *M. varia* | 0.182 | 0.085 |
| 50 | Northeast | *M. hyssopifolia* | 24 | Teno | *M. varia* | 0.216 | 0.099 |
| 50 | Northeast | *M. hyssopifolia* | 25 | Teno | *M. varia* | 0.275 | 0.086 |
| 50 | Northeast | *M. hyssopifolia* | 30 | Teide | *M. lachnophylla* | 0.327 | 0.201 |
| 50 | Northeast | *M. hyssopifolia* | 45 | Southern coast | *M. hyssopifolia* | 0.286 | 0.164 |
| 50 | Northeast | *M. hyssopifolia* | 55 | Southeast | *M. hyssopifolia* | 0.111 | 0.149 |
| 50 | Northeast | *M. hyssopifolia* | 58 | Southeast | *M. hyssopifolia* | 0.127 | 0.165 |
| 50 | Northeast | *M. hyssopifolia* | 59 | Southern coast | *M. hyssopifolia* | 0.106 | 0.167 |
| 50 | Northeast | *M. hyssopifolia* | 60 | Southern coast | *M. hyssopifolia* | 0.322 | 0.143 |
| 50 | Northeast | *M. hyssopifolia* | 61 | Teno | *M. hyssopifolia* | 0.110 | 0.147 |
| 50 | Northeast | *M. hyssopifolia* | 63 | Teno | *M. hyssopifolia* | 0.314 | 0.146 |
| 50 | Northeast | *M. hyssopifolia* | 64 | Teno | *M. hyssopifolia* | 0.058 | 0.154 |
| 50 | Northeast | *M. hyssopifolia* | 65 | Southeast | *M. hyssopifolia* | 0.123 | 0.134 |
| 51 | Adeje | *M. hyssopifolia* | 1 | Southern coast | *M. teneriffae* | 1.170 | 0.145 |
| 51 | Adeje | *M. hyssopifolia* | 3 | Anaga | *M. teneriffae* | 0.717 | 0.347 |
| 51 | Adeje | *M. hyssopifolia* | 5 | Southern coast | *M. teneriffae* | 0.781 | 0.074 |
| 51 | Adeje | *M. hyssopifolia* | 8 | Anaga | *M. rivas-martinezii* | 1.163 | 0.261 |
| 51 | Adeje | *M. hyssopifolia* | 10 | Teide | *M. lasiophylla* | 1.077 | 0.071 |
| 51 | Adeje | *M. hyssopifolia* | 10 | Teide | *M. lasiophylla* | 1.209 | 0.052 |
| 51 | Adeje | *M. hyssopifolia* | 11 | Anaga | *M. varia* | 0.711 | 0.098 |
| 51 | Adeje | *M. hyssopifolia* | 12 | Anaga | *M. varia* | 0.795 | 0.066 |
| 51 | Adeje | *M. hyssopifolia* | 13 | Anaga | *M. varia* | 0.742 | 0.099 |
| 51 | Adeje | *M. hyssopifolia* | 14 | Anaga | *M. varia* | 0.871 | 0.066 |
| 51 | Adeje | *M. hyssopifolia* | 21 | Anaga | *M. varia* | 0.810 | 0.080 |
| 51 | Adeje | *M. hyssopifolia* | 23 | Teno | *M. varia* | 0.554 | 0.067 |
| 51 | Adeje | *M. hyssopifolia* | 24 | Teno | *M. varia* | 0.551 | 0.086 |
| 51 | Adeje | *M. hyssopifolia* | 25 | Teno | *M. varia* | 0.799 | 0.053 |
| 51 | Adeje | *M. hyssopifolia* | 30 | Teide | *M. lachnophylla* | 0.607 | 0.045 |
| 51 | Adeje | *M. hyssopifolia* | 45 | Southern coast | *M. hyssopifolia* | 0.602 | 0.240 |
| 51 | Adeje | *M. hyssopifolia* | 47 | Northeast | *M. hyssopifolia* | 0.794 | 0.053 |
| 51 | Adeje | *M. hyssopifolia* | 48 | Northeast | *M. hyssopifolia* | 0.562 | 0.065 |
| 51 | Adeje | *M. hyssopifolia* | 49 | Northeast | *M. hyssopifolia* | 0.538 | 0.054 |
| 51 | Adeje | *M. hyssopifolia* | 50 | Northeast | *M. hyssopifolia* | 0.367 | 0.078 |
| 51 | Adeje | *M. hyssopifolia* | 52 | Adeje | *M. hyssopifolia* | 0.252 | 0.081 |
| 51 | Adeje | *M. hyssopifolia* | 55 | Southeast | *M. hyssopifolia* | 0.405 | 0.052 |
| 51 | Adeje | *M. hyssopifolia* | 58 | Southeast | *M. hyssopifolia* | 0.329 | 0.104 |
| 51 | Adeje | *M. hyssopifolia* | 59 | Southern coast | *M. hyssopifolia* | 0.451 | 0.308 |
| 51 | Adeje | *M. hyssopifolia* | 60 | Southern coast | *M. hyssopifolia* | 0.556 | 0.108 |
| 51 | Adeje | *M. hyssopifolia* | 61 | Teno | *M. hyssopifolia* | 0.366 | 0.088 |
| 51 | Adeje | *M. hyssopifolia* | 63 | Teno | *M. hyssopifolia* | 0.528 | 0.057 |
| 51 | Adeje | *M. hyssopifolia* | 64 | Teno | *M. hyssopifolia* | 0.259 | 0.060 |
| 51 | Adeje | *M. hyssopifolia* | 65 | Southeast | *M. hyssopifolia* | 0.485 | 0.060 |
| 52 | Adeje | *M. hyssopifolia* | 1 | Southern coast | *M. teneriffae* | 0.769 | 0.095 |
| 52 | Adeje | *M. hyssopifolia* | 3 | Anaga | *M. teneriffae* | 0.369 | 0.161 |
| 52 | Adeje | *M. hyssopifolia* | 5 | Southern coast | *M. teneriffae* | 0.322 | 0.147 |
| 52 | Adeje | *M. hyssopifolia* | 8 | Anaga | *M. rivas-martinezii* | 0.691 | 0.358 |
| 52 | Adeje | *M. hyssopifolia* | 10 | Teide | *M. lasiophylla* | 0.667 | 0.070 |
| 52 | Adeje | *M. hyssopifolia* | 10 | Teide | *M. lasiophylla* | 0.728 | 0.296 |
| 52 | Adeje | *M. hyssopifolia* | 11 | Anaga | *M. varia* | 0.515 | 0.100 |
| 52 | Adeje | *M. hyssopifolia* | 12 | Anaga | *M. varia* | 0.432 | 0.114 |
| 52 | Adeje | *M. hyssopifolia* | 13 | Anaga | *M. varia* | 0.490 | 0.102 |
| 52 | Adeje | *M. hyssopifolia* | 14 | Anaga | *M. varia* | 0.553 | 0.119 |
| 52 | Adeje | *M. hyssopifolia* | 21 | Anaga | *M. varia* | 0.471 | 0.103 |
| 52 | Adeje | *M. hyssopifolia* | 23 | Teno | *M. varia* | 0.203 | 0.111 |
| 52 | Adeje | *M. hyssopifolia* | 24 | Teno | *M. varia* | 0.469 | 0.090 |
| 52 | Adeje | *M. hyssopifolia* | 25 | Teno | *M. varia* | 0.443 | 0.103 |
| 52 | Adeje | *M. hyssopifolia* | 30 | Teide | *M. lachnophylla* | 0.336 | 0.060 |
| 52 | Adeje | *M. hyssopifolia* | 45 | Southern coast | *M. hyssopifolia* | 0.328 | 0.053 |
| 52 | Adeje | *M. hyssopifolia* | 47 | Northeast | *M. hyssopifolia* | 0.438 | 0.060 |
| 52 | Adeje | *M. hyssopifolia* | 48 | Northeast | *M. hyssopifolia* | 0.260 | 0.249 |
| 52 | Adeje | *M. hyssopifolia* | 49 | Northeast | *M. hyssopifolia* | 0.427 | 0.307 |
| 52 | Adeje | *M. hyssopifolia* | 50 | Northeast | *M. hyssopifolia* | 0.246 | 0.113 |
| 52 | Adeje | *M. hyssopifolia* | 55 | Southeast | *M. hyssopifolia* | 0.171 | 0.090 |
| 52 | Adeje | *M. hyssopifolia* | 58 | Southeast | *M. hyssopifolia* | 0.158 | 0.056 |
| 52 | Adeje | *M. hyssopifolia* | 59 | Southern coast | *M. hyssopifolia* | 0.074 | 0.098 |
| 52 | Adeje | *M. hyssopifolia* | 60 | Southern coast | *M. hyssopifolia* | 0.280 | 0.056 |
| 52 | Adeje | *M. hyssopifolia* | 61 | Teno | *M. hyssopifolia* | 0.171 | 0.061 |
| 52 | Adeje | *M. hyssopifolia* | 63 | Teno | *M. hyssopifolia* | 0.347 | 0.080 |
| 52 | Adeje | *M. hyssopifolia* | 64 | Teno | *M. hyssopifolia* | 0.109 | 0.043 |
| 52 | Adeje | *M. hyssopifolia* | 65 | Southeast | *M. hyssopifolia* | 0.244 | 0.061 |
| 55 | Southeast | *M. hyssopifolia* | 1 | Southern coast | *M. teneriffae* | 0.905 | 0.194 |
| 55 | Southeast | *M. hyssopifolia* | 3 | Anaga | *M. teneriffae* | 0.247 | 0.196 |
| 55 | Southeast | *M. hyssopifolia* | 5 | Southern coast | *M. teneriffae* | 0.284 | 0.103 |
| 55 | Southeast | *M. hyssopifolia* | 8 | Anaga | *M. rivas-martinezii* | 0.539 | 0.194 |
| 55 | Southeast | *M. hyssopifolia* | 10 | Teide | *M. lasiophylla* | 0.640 | 0.124 |
| 55 | Southeast | *M. hyssopifolia* | 10 | Teide | *M. lasiophylla* | 0.699 | 0.118 |
| 55 | Southeast | *M. hyssopifolia* | 11 | Anaga | *M. varia* | 0.422 | 0.197 |
| 55 | Southeast | *M. hyssopifolia* | 12 | Anaga | *M. varia* | 0.355 | 0.144 |
| 55 | Southeast | *M. hyssopifolia* | 13 | Anaga | *M. varia* | 0.380 | 0.274 |
| 55 | Southeast | *M. hyssopifolia* | 14 | Anaga | *M. varia* | 0.529 | 0.334 |
| 55 | Southeast | *M. hyssopifolia* | 21 | Anaga | *M. varia* | 0.365 | 0.185 |
| 55 | Southeast | *M. hyssopifolia* | 23 | Teno | *M. varia* | 0.152 | 0.102 |
| 55 | Southeast | *M. hyssopifolia* | 24 | Teno | *M. varia* | 0.415 | 0.141 |
| 55 | Southeast | *M. hyssopifolia* | 25 | Teno | *M. varia* | 0.211 | 0.082 |
| 55 | Southeast | *M. hyssopifolia* | 30 | Teide | *M. lachnophylla* | 0.270 | 0.135 |
| 55 | Southeast | *M. hyssopifolia* | 58 | Southeast | *M. hyssopifolia* | 0.148 | 0.101 |
| 55 | Southeast | *M. hyssopifolia* | 61 | Teno | *M. hyssopifolia* | 0.105 | 0.109 |
| 55 | Southeast | *M. hyssopifolia* | 63 | Teno | *M. hyssopifolia* | 0.301 | 0.121 |
| 55 | Southeast | *M. hyssopifolia* | 64 | Teno | *M. hyssopifolia* | 0.108 | 0.103 |
| 55 | Southeast | *M. hyssopifolia* | 65 | Southeast | *M. hyssopifolia* | 0.095 | 0.099 |
| 58 | Southeast | *M. hyssopifolia* | 1 | Southern coast | *M. teneriffae* | 0.843 | 0.137 |
| 58 | Southeast | *M. hyssopifolia* | 3 | Anaga | *M. teneriffae* | 0.341 | 0.097 |
| 58 | Southeast | *M. hyssopifolia* | 5 | Southern coast | *M. teneriffae* | 0.278 | 0.130 |
| 58 | Southeast | *M. hyssopifolia* | 8 | Anaga | *M. rivas-martinezii* | 0.580 | 0.118 |
| 58 | Southeast | *M. hyssopifolia* | 10 | Teide | *M. lasiophylla* | 0.554 | 0.095 |
| 58 | Southeast | *M. hyssopifolia* | 10 | Teide | *M. lasiophylla* | 0.656 | 0.096 |
| 58 | Southeast | *M. hyssopifolia* | 11 | Anaga | *M. varia* | 0.505 | 0.103 |
| 58 | Southeast | *M. hyssopifolia* | 12 | Anaga | *M. varia* | 0.376 | 0.188 |
| 58 | Southeast | *M. hyssopifolia* | 13 | Anaga | *M. varia* | 0.419 | 0.177 |
| 58 | Southeast | *M. hyssopifolia* | 14 | Anaga | *M. varia* | 0.510 | 0.168 |
| 58 | Southeast | *M. hyssopifolia* | 21 | Anaga | *M. varia* | 0.452 | 0.104 |
| 58 | Southeast | *M. hyssopifolia* | 23 | Teno | *M. varia* | 0.277 | 0.214 |
| 58 | Southeast | *M. hyssopifolia* | 24 | Teno | *M. varia* | 0.334 | 0.135 |
| 58 | Southeast | *M. hyssopifolia* | 25 | Teno | *M. varia* | 0.380 | 0.072 |
| 58 | Southeast | *M. hyssopifolia* | 30 | Teide | *M. lachnophylla* | 0.278 | 0.108 |
| 58 | Southeast | *M. hyssopifolia* | 61 | Teno | *M. hyssopifolia* | 0.185 | 0.117 |
| 58 | Southeast | *M. hyssopifolia* | 63 | Teno | *M. hyssopifolia* | 0.367 | 0.111 |
| 58 | Southeast | *M. hyssopifolia* | 64 | Teno | *M. hyssopifolia* | 0.156 | 0.093 |
| 58 | Southeast | *M. hyssopifolia* | 65 | Southeast | *M. hyssopifolia* | 0.116 | 0.109 |
| 59 | Southern coast | *M. hyssopifolia* | 1 | Southern coast | *M. teneriffae* | 0.657 | 0.184 |
| 59 | Southern coast | *M. hyssopifolia* | 3 | Anaga | *M. teneriffae* | 0.249 | 0.322 |
| 59 | Southern coast | *M. hyssopifolia* | 5 | Southern coast | *M. teneriffae* | 0.259 | 0.086 |
| 59 | Southern coast | *M. hyssopifolia* | 8 | Anaga | *M. rivas-martinezii* | 0.581 | 0.265 |
| 59 | Southern coast | *M. hyssopifolia* | 10 | Teide | *M. lasiophylla* | 0.570 | 0.238 |
| 59 | Southern coast | *M. hyssopifolia* | 10 | Teide | *M. lasiophylla* | 0.585 | 0.156 |
| 59 | Southern coast | *M. hyssopifolia* | 11 | Anaga | *M. varia* | 0.467 | 0.116 |
| 59 | Southern coast | *M. hyssopifolia* | 12 | Anaga | *M. varia* | 0.280 | 0.079 |
| 59 | Southern coast | *M. hyssopifolia* | 13 | Anaga | *M. varia* | 0.447 | 0.113 |
| 59 | Southern coast | *M. hyssopifolia* | 14 | Anaga | *M. varia* | 0.392 | 0.102 |
| 59 | Southern coast | *M. hyssopifolia* | 21 | Anaga | *M. varia* | 0.299 | 0.092 |
| 59 | Southern coast | *M. hyssopifolia* | 23 | Teno | *M. varia* | 0.087 | 0.088 |
| 59 | Southern coast | *M. hyssopifolia* | 24 | Teno | *M. varia* | 0.261 | 0.109 |
| 59 | Southern coast | *M. hyssopifolia* | 25 | Teno | *M. varia* | 0.198 | 0.081 |
| 59 | Southern coast | *M. hyssopifolia* | 30 | Teide | *M. lachnophylla* | 0.131 | 0.218 |
| 59 | Southern coast | *M. hyssopifolia* | 55 | Southeast | *M. hyssopifolia* | 0.098 | 0.206 |
| 59 | Southern coast | *M. hyssopifolia* | 58 | Southeast | *M. hyssopifolia* | 0.036 | 0.176 |
| 59 | Southern coast | *M. hyssopifolia* | 60 | Southern coast | *M. hyssopifolia* | 0.186 | 0.175 |
| 59 | Southern coast | *M. hyssopifolia* | 61 | Teno | *M. hyssopifolia* | 0.138 | 0.214 |
| 59 | Southern coast | *M. hyssopifolia* | 63 | Teno | *M. hyssopifolia* | 0.296 | 0.147 |
| 59 | Southern coast | *M. hyssopifolia* | 64 | Teno | *M. hyssopifolia* | 0.102 | 0.215 |
| 59 | Southern coast | *M. hyssopifolia* | 65 | Southeast | *M. hyssopifolia* | 0.100 | 0.216 |
| 60 | Southern coast | *M. hyssopifolia* | 1 | Southern coast | *M. teneriffae* | 0.777 | 0.314 |
| 60 | Southern coast | *M. hyssopifolia* | 3 | Anaga | *M. teneriffae* | 0.235 | 0.156 |
| 60 | Southern coast | *M. hyssopifolia* | 5 | Southern coast | *M. teneriffae* | 0.448 | 0.331 |
| 60 | Southern coast | *M. hyssopifolia* | 8 | Anaga | *M. rivas-martinezii* | 0.731 | 0.074 |
| 60 | Southern coast | *M. hyssopifolia* | 10 | Teide | *M. lasiophylla* | 0.909 | 0.151 |
| 60 | Southern coast | *M. hyssopifolia* | 10 | Teide | *M. lasiophylla* | 0.993 | 0.124 |
| 60 | Southern coast | *M. hyssopifolia* | 11 | Anaga | *M. varia* | 0.380 | 0.172 |
| 60 | Southern coast | *M. hyssopifolia* | 12 | Anaga | *M. varia* | 0.413 | 0.106 |
| 60 | Southern coast | *M. hyssopifolia* | 13 | Anaga | *M. varia* | 0.440 | 0.150 |
| 60 | Southern coast | *M. hyssopifolia* | 14 | Anaga | *M. varia* | 0.527 | 0.086 |
| 60 | Southern coast | *M. hyssopifolia* | 21 | Anaga | *M. varia* | 0.472 | 0.094 |
| 60 | Southern coast | *M. hyssopifolia* | 23 | Teno | *M. varia* | 0.274 | 0.127 |
| 60 | Southern coast | *M. hyssopifolia* | 24 | Teno | *M. varia* | 0.354 | 0.109 |
| 60 | Southern coast | *M. hyssopifolia* | 25 | Teno | *M. varia* | 0.484 | 0.109 |
| 60 | Southern coast | *M. hyssopifolia* | 30 | Teide | *M. lachnophylla* | 0.369 | 0.143 |
| 60 | Southern coast | *M. hyssopifolia* | 55 | Southeast | *M. hyssopifolia* | 0.296 | 0.084 |
| 60 | Southern coast | *M. hyssopifolia* | 58 | Southeast | *M. hyssopifolia* | 0.245 | 0.089 |
| 60 | Southern coast | *M. hyssopifolia* | 61 | Teno | *M. hyssopifolia* | 0.312 | 0.142 |
| 60 | Southern coast | *M. hyssopifolia* | 63 | Teno | *M. hyssopifolia* | 0.382 | 0.133 |
| 60 | Southern coast | *M. hyssopifolia* | 64 | Teno | *M. hyssopifolia* | 0.220 | 0.085 |
| 60 | Southern coast | *M. hyssopifolia* | 65 | Southeast | *M. hyssopifolia* | 0.234 | 0.094 |
| 61 | Teno | *M. hyssopifolia* | 1 | Southern coast | *M. teneriffae* | 0.816 | 0.078 |
| 61 | Teno | *M. hyssopifolia* | 3 | Anaga | *M. teneriffae* | 0.294 | 0.073 |
| 61 | Teno | *M. hyssopifolia* | 5 | Southern coast | *M. teneriffae* | 0.374 | 0.277 |
| 61 | Teno | *M. hyssopifolia* | 8 | Anaga | *M. rivas-martinezii* | 0.658 | 0.144 |
| 61 | Teno | *M. hyssopifolia* | 10 | Teide | *M. lasiophylla* | 0.727 | 0.102 |
| 61 | Teno | *M. hyssopifolia* | 10 | Teide | *M. lasiophylla* | 0.836 | 0.223 |
| 61 | Teno | *M. hyssopifolia* | 11 | Anaga | *M. varia* | 0.459 | 0.357 |
| 61 | Teno | *M. hyssopifolia* | 12 | Anaga | *M. varia* | 0.356 | 0.184 |
| 61 | Teno | *M. hyssopifolia* | 13 | Anaga | *M. varia* | 0.300 | 0.109 |
| 61 | Teno | *M. hyssopifolia* | 14 | Anaga | *M. varia* | 0.329 | 0.135 |
| 61 | Teno | *M. hyssopifolia* | 21 | Anaga | *M. varia* | 0.383 | 0.073 |
| 61 | Teno | *M. hyssopifolia* | 23 | Teno | *M. varia* | 0.090 | 0.094 |
| 61 | Teno | *M. hyssopifolia* | 24 | Teno | *M. varia* | 0.278 | 0.126 |
| 61 | Teno | *M. hyssopifolia* | 25 | Teno | *M. varia* | 0.161 | 0.090 |
| 61 | Teno | *M. hyssopifolia* | 30 | Teide | *M. lachnophylla* | 0.277 | 0.183 |
| 61 | Teno | *M. hyssopifolia* | 63 | Teno | *M. hyssopifolia* | 0.238 | 0.216 |
| 61 | Teno | *M. hyssopifolia* | 64 | Teno | *M. hyssopifolia* | 0.066 | 0.216 |
| 63 | Teno | *M. hyssopifolia* | 1 | Southern coast | *M. teneriffae* | 0.997 | 0.151 |
| 63 | Teno | *M. hyssopifolia* | 3 | Anaga | *M. teneriffae* | 0.518 | 0.083 |
| 63 | Teno | *M. hyssopifolia* | 5 | Southern coast | *M. teneriffae* | 0.583 | 0.131 |
| 63 | Teno | *M. hyssopifolia* | 8 | Anaga | *M. rivas-martinezii* | 0.856 | 0.123 |
| 63 | Teno | *M. hyssopifolia* | 10 | Teide | *M. lasiophylla* | 0.878 | 0.110 |
| 63 | Teno | *M. hyssopifolia* | 10 | Teide | *M. lasiophylla* | 0.795 | 0.109 |
| 63 | Teno | *M. hyssopifolia* | 11 | Anaga | *M. varia* | 0.511 | 0.111 |
| 63 | Teno | *M. hyssopifolia* | 12 | Anaga | *M. varia* | 0.457 | 0.188 |
| 63 | Teno | *M. hyssopifolia* | 13 | Anaga | *M. varia* | 0.609 | 0.187 |
| 63 | Teno | *M. hyssopifolia* | 14 | Anaga | *M. varia* | 0.643 | 0.179 |
| 63 | Teno | *M. hyssopifolia* | 21 | Anaga | *M. varia* | 0.598 | 0.118 |
| 63 | Teno | *M. hyssopifolia* | 23 | Teno | *M. varia* | 0.293 | 0.197 |
| 63 | Teno | *M. hyssopifolia* | 24 | Teno | *M. varia* | 0.419 | 0.118 |
| 63 | Teno | *M. hyssopifolia* | 25 | Teno | *M. varia* | 0.338 | 0.105 |
| 63 | Teno | *M. hyssopifolia* | 30 | Teide | *M. lachnophylla* | 0.370 | 0.116 |
| 63 | Teno | *M. hyssopifolia* | 64 | Teno | *M. hyssopifolia* | 0.270 | 0.101 |
| 64 | Teno | *M. hyssopifolia* | 1 | Southern coast | *M. teneriffae* | 1.017 | 0.101 |
| 64 | Teno | *M. hyssopifolia* | 3 | Anaga | *M. teneriffae* | 0.291 | 0.184 |
| 64 | Teno | *M. hyssopifolia* | 5 | Southern coast | *M. teneriffae* | 0.506 | 0.131 |
| 64 | Teno | *M. hyssopifolia* | 8 | Anaga | *M. rivas-martinezii* | 0.824 | 0.287 |
| 64 | Teno | *M. hyssopifolia* | 10 | Teide | *M. lasiophylla* | 0.745 | 0.094 |
| 64 | Teno | *M. hyssopifolia* | 10 | Teide | *M. lasiophylla* | 0.838 | 0.297 |
| 64 | Teno | *M. hyssopifolia* | 11 | Anaga | *M. varia* | 0.418 | 0.081 |
| 64 | Teno | *M. hyssopifolia* | 12 | Anaga | *M. varia* | 0.426 | 0.111 |
| 64 | Teno | *M. hyssopifolia* | 13 | Anaga | *M. varia* | 0.405 | 0.115 |
| 64 | Teno | *M. hyssopifolia* | 14 | Anaga | *M. varia* | 0.506 | 0.104 |
| 64 | Teno | *M. hyssopifolia* | 21 | Anaga | *M. varia* | 0.448 | 0.093 |
| 64 | Teno | *M. hyssopifolia* | 23 | Teno | *M. varia* | 0.242 | 0.109 |
| 64 | Teno | *M. hyssopifolia* | 24 | Teno | *M. varia* | 0.305 | 0.093 |
| 64 | Teno | *M. hyssopifolia* | 25 | Teno | *M. varia* | 0.329 | 0.103 |
| 64 | Teno | *M. hyssopifolia* | 30 | Teide | *M. lachnophylla* | 0.387 | 0.093 |
| 65 | Southeast | *M. hyssopifolia* | 1 | Southern coast | *M. teneriffae* | 1.001 | 0.126 |
| 65 | Southeast | *M. hyssopifolia* | 3 | Anaga | *M. teneriffae* | 0.289 | 0.127 |
| 65 | Southeast | *M. hyssopifolia* | 5 | Southern coast | *M. teneriffae* | 0.241 | 0.117 |
| 65 | Southeast | *M. hyssopifolia* | 8 | Anaga | *M. rivas-martinezii* | 0.487 | 0.098 |
| 65 | Southeast | *M. hyssopifolia* | 10 | Teide | *M. lasiophylla* | 0.692 | 0.159 |
| 65 | Southeast | *M. hyssopifolia* | 10 | Teide | *M. lasiophylla* | 0.547 | 0.093 |
| 65 | Southeast | *M. hyssopifolia* | 11 | Anaga | *M. varia* | 0.465 | 0.120 |
| 65 | Southeast | *M. hyssopifolia* | 12 | Anaga | *M. varia* | 0.402 | 0.101 |
| 65 | Southeast | *M. hyssopifolia* | 13 | Anaga | *M. varia* | 0.454 | 0.108 |
| 65 | Southeast | *M. hyssopifolia* | 14 | Anaga | *M. varia* | 0.496 | 0.128 |
| 65 | Southeast | *M. hyssopifolia* | 21 | Anaga | *M. varia* | 0.509 | 0.091 |
| 65 | Southeast | *M. hyssopifolia* | 23 | Teno | *M. varia* | 0.165 | 0.161 |
| 65 | Southeast | *M. hyssopifolia* | 24 | Teno | *M. varia* | 0.383 | 0.133 |
| 65 | Southeast | *M. hyssopifolia* | 25 | Teno | *M. varia* | 0.262 | 0.115 |
| 65 | Southeast | *M. hyssopifolia* | 30 | Teide | *M. lachnophylla* | 0.225 | 0.114 |
| 65 | Southeast | *M. hyssopifolia* | 61 | Teno | *M. hyssopifolia* | 0.173 | 0.342 |
| 65 | Southeast | *M. hyssopifolia* | 63 | Teno | *M. hyssopifolia* | 0.228 | 0.357 |
| 65 | Southeast | *M. hyssopifolia* | 64 | Teno | *M. hyssopifolia* | 0.219 | 0.185 |
